# Glyceraldehyde May Induce the Generation of High-Molecular-Weight Species of HSP90β and Autophagy-Related Proteins and Alter Protein Levels in Human Renal Epithelial Cells

**DOI:** 10.3390/biom16101488

**Published:** 2026-10-13

**Authors:** Thao Thanh Nguyen, Takanobu Takata, Shinya Inoue, Dung Anh Vu, Akihiro Shioya, Miyako Shimasaki, Motona Kumagai, Jia Han, Yao Liu, Wenpeng Jiao, Mitsuaki Yoshida, Yasuhisa Sawai, Hiroyuki Nakano, Togen Masauji, Katsuhito Miyazawa, Sohsuke Yamada

**Affiliations:** 1 Department of Pathology and Laboratory Medicine, Kanazawa Medical University, Uchinada 920-0293, Ishikawa, Japan; nguyenthanhthao121997@gmail.com (T.T.N.); surgeonvuanhdung@gmail.com (D.A.V.); a-shioya@kanazawa-med.ac.jp (A.S.); hanjia0227@yahoo.co.jp (J.H.); liuyao5139@163.com (Y.L.); jiaowenpeng163@163.com (W.J.); mi-yoshi@kanazawa-med.ac.jp (M.Y.); 2 Department of Pathology, Kanazawa Medical University Hospital, Uchinada 920-0293, Ishikawa, Japan; 3 Department of Pathophysiological and Experimental Pathology, Kanazawa Medical University, Uchinada 920-0293, Ishikawa, Japan; miya0807@kanazawa-med.ac.jp (M.S.); kumagaim@kanazawa-med.ac.jp (M.K.); 4 Department of Pharmacy, Kanazawa Medical University Hospital, Uchinada 920-0293, Ishikawa, Japan; masauji@kanazawa-med.ac.jp; 5 Department of Urology, Kanazawa Medical University, Uchinada 920-0293, Ishikawa, Japan; s-inoue@kanazawa-med.ac.jp (S.I.); miyazawa@kanazawa-med.ac.jp (K.M.); 6 Department of Oncogenic Pathology, Kanazawa Medical University, Uchinada 920-0293, Ishikawa, Japan; 7 Department of Oral and Maxillofacial Surgery, Kanazawa Medical University, Uchinada 920-0293, Ishikawa, Japan; y-sawai@kanazawa-med.ac.jp (Y.S.); nakano-h@kanazawa-med.ac.jp (H.N.)

**Keywords:** kidney disease, renal epithelial cell, glyceraldehyde, argpyrimidine, advanced glycation end-product, heat shock protein 90, Beclin-1, ATG7, microtubule-associated protein 1 light chain 3, p62

## Abstract

Kidney disease (KD) is a major health problem, and metabolic disorders contribute to diabetic nephropathy and chronic kidney disease, in which renal epithelial cell dysfunction plays an important role. Intracellular advanced glycation end-products (AGEs) may contribute to KD progression by disrupting cellular homeostasis; however, the underlying mechanisms remain unclear. Therefore, we aimed to investigate the effects of glyceraldehyde (GA) on molecular chaperone- and autophagy-related proteins in human renal epithelial cells (HK-2), with particular attention to AGE-related processes. Administration of high-fructose corn syrup to healthy Wistar rats increased argpyrimidine immunoreactivity in the renal tubular epithelial cells, compared with that in controls, suggesting enhanced accumulation of this GA-related AGE in vivo. In HK-2 cells, GA treatment caused concentration-dependent reductions in NADPH and ATP levels, accompanied by morphological changes. High molecular weight (HMW) immunoreactive species of HSP90β, Beclin-1, and ATG7 appeared following GA treatment. GA at 1.5 mM increased LC3-I, LC3-II, and p62 levels, whereas treatment with 2 mM GA attenuated these increases. Aminoguanidine, an inhibitor of AGE formation, attenuated these GA-induced alterations. Overall, GA may induce the generation of HMW species of HSP90β and autophagy-related proteins and may contribute to altered cellular homeostasis, with AGE-related processes potentially contributing to these effects.

## 1. Introduction

Kidney disease is a serious global health issue [1,2,3]. Metabolic and lifestyle-related factors contribute to the development and progression of diabetic nephropathy (DN) and chronic kidney disease (CKD), which are closely associated with diabetes mellitus and other metabolic disorders [4,5,6]. Kidney tissue comprises various cell types; for instance, renal tubular epithelial cells regulate the reabsorption of minerals, glucose, amino acids, and water from the glomerular filtrate, thereby determining urine volume [7,8,9]; in humans, their dysfunction may substantially affect renal function. Further, renal tubular epithelial cell dysfunction contributes to the development and progression of DN and CKD [7,8,9]. Advanced glycation end products (AGEs) are generated from saccharides, including glucose, fructose, and ribose, as well as their metabolic intermediates or by-products, such as glyceraldehyde (GA), methylglyoxal (MG), and glyoxal [10,11,12,13]. Since the early 20th century, various AGEs have been identified, including N^ε^-carboxymethyl-lysine (CML), N^ε^-carboxyethyl-lysine, N^δ^-(5-hydroxy-5-methyl-4-imidazolone-2-yl)-ornithine (MG-derived hydroimidazolone; MG-H1), and argpyrimidine [10,11,12,13]. Serum AGE levels have been shown to increase in patients with DN [14]. Takahashi et al. reported that CML-modified proteins accumulated in proximal tubular epithelial (PTE) cells in the kidney tissue of diabetic model mice and that CML accumulation may suppress autophagy [15]. Furthermore, they demonstrated that autophagy deficiency caused by *ATG5* knockout promotes intracellular AGE accumulation in PTE cells through impaired lysosomal degradation [15]. In addition, AGE-modified bovine serum albumin (BSA; AGE-BSA), a model of dietary and circulating AGEs, has been shown to suppress autophagy in human renal epithelial cells (HK-2) [16]. However, based on these findings [15], this effect may partly result from the uptake of extracellular AGEs into PTE cells.

GA is a highly reactive metabolic intermediate that can contribute to AGE formation [17,18]. Several metabolic pathways may contribute to the intracellular generation of GA, including (i) fructose metabolism, in which fructose is converted to fructose-1-phosphate by fructokinase and subsequently cleaved by aldolase B to generate GA; (ii) the polyol pathway, in which glucose is converted to sorbitol by aldose reductase and then to fructose by sorbitol dehydrogenase, after which fructose can enter fructose metabolism and contribute to GA generation; and (iii) glycolysis, in which glyceraldehyde-3-phosphate is generated from glucose and can contribute to GA formation under conditions favoring its alternative metabolism [19]. Key enzymes involved in these metabolic pathways are expressed in the human kidney, including the renal tubules, indicating that renal tubular epithelial cells possess the metabolic machinery involved in glucose and fructose metabolism and may therefore have the potential to generate GA intracellularly [20,21]. The intracellularly generated GA may subsequently undergo non-enzymatic reactions with intracellular proteins and contribute to the formation of GA-derived AGEs (GA-AGEs), providing a potential mechanism for intracellular GA-AGE formation in human renal tubular epithelial cells [19].

Among the intracellular proteins that may be affected by GA are proteins involved in cellular homeostasis, such as molecular chaperones and autophagy-related proteins. Heat shock protein 90 (HSP90), Beclin-1, ATG7, microtubule-associated protein 1 light chain 3 (LC3), and p62 play important roles in maintaining cellular homeostasis through molecular chaperone activity and autophagy regulation [22,23,24,25]. HSP90 has two isoforms, HSP90α and HSP90β, which work as molecular chaperones involved in protein quality control [26]. In addition, HSP90 participates in autophagy regulation through its interactions with Beclin-1 during early autophagosome generation in PTE cells [22]. Beclin-1, ATG7, LC3, and p62 are involved in different stages of autophagy, including autophagosome formation, cargo sequestration, and degradation [23,24,25]. However, whether GA induces changes in HSP90 and autophagy-related proteins in HK-2 cells remains unclear.

Therefore, in this study, we aimed to investigate whether argpyrimidine accumulation is increased in the kidney tissue of healthy Wistar rats after oral administration of high-fructose corn syrup (HFCS) for 8 weeks. Furthermore, we hypothesized that GA may induce changes in HSP90 and autophagy-related proteins and that these changes may contribute to altered cellular homeostasis in HK-2 cells.

## 2. Materials and Methods

### 2.1. Animals and Experimental Design

All experiments using rats were approved by the Institutional Animal Care and Use Committee of Kanazawa Medical University (clearance number: 2017-59; approval date: 1 April 2017) and were conducted in accordance with the Institutional Animal Care and Use Guidelines (“Basic Guidelines for the Conduct of Animal Experiments” and “Basic Guidelines for Animal Care Management”). Male Wistar rats (11 weeks old; n = 20) were obtained from SLC Japan, Inc. (Hamamatsu, Japan). After 1 week of acclimation, the rats were randomly divided into two groups (control and HFCS, n = 10 animals each). The experimental unit was an individual rat. The animals were housed individually and had *ad libitum* access to a standard feed (Labo MR stock, Nosan, Japan) and commercially available water (Évian-les-Bains, France). In the HFCS group, a commercial HFCS-55 (composed of 41.25 g fructose, 33.75 g glucose, and 25.0 g water per 100 g syrup, with a density of 1.37 g/mL and an energy content of 276 kcal/100 g) was added to the drinking water at a final concentration of 137 g/L, corresponding to 10% (*v*/*v*) HFCS-55. The rats were maintained under controlled room temperature (RT; 24 ± 3 °C) and humidity (50 ± 20%) with a 12 h light/dark cycle. The experimental treatment was initiated at 12 weeks of age and continued for 8 weeks. At 20 weeks of age, the rats were anesthetized via isoflurane inhalation, and tissues were collected under deep anesthesia without recovery.

### 2.2. Reagents and Antibodies

HK-2 cells (Cat. CRL-2190) were obtained from the American Type Culture Collection (Manassas, VA, USA). GA (Cat. 17014-81), Blocking One (Cat. 03953-95), Western blot (WB) stripping solution (Cat. 05364-55), ethanol (Cat. 14713-53), 4% paraformaldehyde phosphate buffer solution (Cat. 09154-14), and skim milk (Cat. 31149-75) were purchased from Nacalai Tesque (Kyoto, Japan). Aminoguanidine (AG; Cat. 328-26432), ImmunoStar LD kits (Cat. 292-69903), methanol (Cat. 138-01836), xylene (Cat. 244-00081), 0.5% eosin Y-ethanol solution (Cat. 051-06495), and Mayer’s hematoxylin solution (Cat. 131-09663) were obtained from FUJIFILM Wako Pure Chemical Corporation (Osaka, Japan). Dulbecco’s modified Eagle’s medium (DMEM)/Nutrient Mixture F-12 Ham (DMEM/F-12 Ham; Cat. D8437-500ML) and penicillin/streptomycin were purchased from Sigma-Aldrich Co. LLC (St. Louis, MO, USA). Fetal bovine serum (FBS; South American origin; Cat. FB-1001/500) was obtained from Biosera (Maine-et-Loire, France). Cell Counting Kit-8 (WST-8 assay) kit (Cat. 347-07621) was purchased from Dojindo Laboratories (Kumamoto, Japan). CellTiter-Glo Luminescent Cell Viability Assay (Cat. G7570) was obtained from Promega (Madison, WI, USA). Radioimmunoprecipitation assay (RIPA) buffer (Cat. 89900), bicinchoninic acid (BCA) protein assay kits (Cat. 23209), 96-well microplates (Cat. 167008 and 165306), glass chamber slides (Cat. 154526), HRP-linked goat anti-mouse antibody (Cat. 31432) and HRP-linked donkey anti-rabbit IgG antibody (Cat. 31458) were obtained from Thermo Fisher Scientific Inc. (Waltham, MA, USA). Sixty-mm culture dishes were obtained from Becton Dickinson (Franklin Lakes, NJ, USA). Ethylenediamine-N,N,N′,N′-tetraacetic acid (EDTA)-free protease inhibitor cocktail was obtained from Roche Applied Science (Penzberg, Germany). Prestained Protein Marker (Cat. BNPM41) was obtained from BioNexus (Oakland, CA, USA). Precision Plus Protein Dual Xtra Standards (Cat. 161-0377), Mini-PROTEAN TGX Precast gels (Cat. 456-1083), Laemmli sample buffer (Cat. 161-0737), and Tris/glycine/sodium dodecyl sulfate (SDS) buffer (Cat. 161-0772) were obtained from Bio-Rad Laboratories (Hercules, CA, USA). Polyvinylidene difluoride (PVDF) membranes (0.45 μm) were obtained from MilliporeSigma (Burlington, MA, USA). Sodium citrate antigen retrieval buffer (Cat. PR30001) was obtained from Proteintech (Rosemont, IL, USA). Histofine Simple Stain MAX-PO (Multi) (Cat. 424152) and 3,3′-diaminobenzidine tetrahydrochloride (DAB) (Cat. 425011) were obtained from Nichirei Biosciences Inc. (Tokyo, Japan). Multi Mount 480 (Cat. 5901) and NEO micro cover glass (25 × 60 mm) were purchased from Matsunami Glass Ind., Ltd. (Osaka, Japan). Mouse anti-MG monoclonal antibody (MMG-030n) was obtained from JaICA (Shizuoka, Japan). Anti-Beclin-1 antibody (ab207612), anti-ATG7 antibody (ab133528), anti-HSP90α antibody (ab2928), anti-HSP90β antibody (ab119833), anti-HSP90 antibody (ab109248), anti-p62 antibody (ab207305), and anti-β-actin antibody (ab3280) were purchased from Abcam plc. (Cambridge, UK). Anti-LC3 antibody (Cat. 12741) and anti-p62 antibody (Cat. 5114) were obtained from Cell Signaling Technology (Danvers, MA, USA). All other reagents and chemicals were purchased from FUJIFILM Wako Pure Chemical Corporation (Osaka, Japan).

### 2.3. Hematoxylin and Eosin (H&E) Staining of Kidney Sections

Kidney tissue samples from the control and HFCS groups were fixed in 10% formalin, embedded in paraffin, and cut into 3-µm-thick sections. After deparaffinization in xylene and rehydration through graded ethanol, the kidney sections were stained with H&E, dehydrated through graded ethanol, cleared in xylene, mounted with Multi Mount 480, and scanned using a NanoZoomer slide scanner (Hamamatsu Photonics K.K., Hamamatsu, Japan).

### 2.4. Immunohistochemical Staining of Kidney Sections

Paraffin-embedded kidney tissues were cut to a thickness of 3 μm and immunohistochemically stained for argpyrimidine and p62. After deparaffinization in xylene and rehydration through graded ethanol, heat-induced antigen retrieval was performed by incubating sections in citrate buffer (pH 6.0) at 95 °C for 20 min, followed by cooling at RT for 20 min. Phosphate-buffered saline without Ca^2+^ and Mg^2+^ [PBS(-)] was used as the buffer for antibody dilution and washing throughout the immunohistochemical procedure. Endogenous peroxidase activity was blocked with 3% hydrogen peroxide in PBS(-) for 10 min. Tissue sections were then incubated with Blocking One solution (1:10) for 1 h at RT. Sections were incubated overnight at 4 °C with either mouse anti-MG monoclonal antibody (1:200) for argpyrimidine detection or anti-p62 antibody (Cat. 5114; 1:100) for p62 detection. After washing three times with PBS(-), sections were incubated with Histofine Simple Stain MAX-PO (Multi) reagent according to the manufacturer’s instructions. After washing with PBS(-), immunoreactivity was visualized using DAB for 2 min according to the manufacturer’s instructions. The sections were counterstained with hematoxylin for 2 min and rinsed in running tap water. The sections were dehydrated through graded ethanol (70%, 80%, 90%, and 100%), cleared with xylene, mounted with Multi Mount 480, and observed using the NanoZoomer slide scanner (Hamamatsu Photonics K.K.). Mouse liver tissue was used as a positive control. All tissue sections were reviewed under blinded conditions by two pathologists together with two scientific investigators to determine the percentage of positively stained area. The interobserver agreement, assessed using the intraclass correlation coefficient, exceeded 0.85. Cases with discordant assessments, accounting for <15% of the total, were re-examined by a senior pathologist to establish a final consensus.

No animals were excluded from this study, and kidney tissues were collected from all rats. However, the number of tissue sections available for final histological and immunohistochemical evaluation varied among the different analyses. Some sections were used during preliminary optimization of the staining protocols, and the availability of suitable sections for final evaluation therefore differed among the staining analyses. Consequently, the number of evaluable specimens varied among the different histological and immunohistochemical analyses.

### 2.5. Cell Culture and Seeding

HK-2 cells were cultured in DMEM/F-12 Ham supplemented with 10% FBS, 100 U/mL penicillin, and 100 µg/mL streptomycin under standard cell culture conditions (humidified atmosphere, 5% CO_2_, and 37 °C). Cells at passages 3–5 were used for all experiments and seeded at a density of 3.8 × 10^4^ cells/cm^2^ on 96-well microplates, glass chamber slides, and 60-mm culture dishes for further analysis.

### 2.6. Assessment of Nicotinamide Adenine Dinucleotide Phosphate (NADPH) and Adenosine Triphosphate (ATP) Levels in HK-2 Cells

HK-2 cells were cultured in two types of 96-well microplates: Cat. 167008 for the WST-8 assay and Cat. 165306 for the CellTiter-Glo Luminescent Cell Viability assay. Prior to treatment, GA and AG were individually dissolved in PBS(-) and filtered. The final volume of PBS(-) in the culture medium was adjusted to 2.0 μL/100 μL medium, including the volume of the GA or AG solution. For GA treatment experiments, 24 h after cell seeding, cells were treated with 0, 0.5, 1, 1.5, and 2 mM GA for 24 h. For AG pretreatment experiments, 24 h after cell seeding, cells were pretreated with 0 or 8 mM AG for 2 h. After pretreatment with AG, cells were exposed to 0, 1.5, and 2 mM GA for an additional 24 h. The WST-8 assay, which reflects NADPH-dependent cellular reducing activity through the reduction of WST-8 to formazan, was performed according to the manufacturer’s instructions. Absorbance was measured using an iMark microplate reader (Bio-Rad Laboratories). The CellTiter-Glo Luminescent Cell Viability assay, which measures intracellular ATP levels, was performed according to the manufacturer’s instructions. Briefly, cells were incubated with CellTiter-Glo reagent for 10 min, and luminescence was measured using a 96-well microplate reader (GloMax-96 microplate luminometer, Promega). Background luminescence from the culture medium without cells was subtracted from the experimental values.

### 2.7. Phase-Contrast Microscopy of HK-2 Cells

HK-2 cells cultured in 60-mm dishes were used for phase-contrast microscopic observation. GA treatment and AG pretreatment were performed under the same conditions as those described in Section 2.6. Microscopic observations were performed using an ALL-IN-ONE fluorescence microscope (BZ-X700; KEYENCE, Osaka, Japan). A 10× objective lens was used in phase-contrast mode, and the exposure time was set to 1.2 s.

### 2.8. H&E Staining of HK-2 Cells

HK-2 cells cultured on glass chamber slides were used for H&E staining. For GA treatment experiments, 24 h after seeding on glass chamber slides, cells were treated with 0, 1, 1.5, and 2 mM GA for 24 h. For the AG-pretreatment experiments, cells were pretreated with 0 or 8 mM AG for 2 h and subsequently exposed to 0, 1.5, and 2 mM GA for an additional 24 h. Two four-well chamber slides were used to accommodate all treatment conditions, and the experiment was independently repeated four times. After treatment, cells were washed with PBS(-), fixed with 4% paraformaldehyde, and stained with H&E according to standard procedures. Cellular morphology was observed using the NanoZoomer slide scanner.

### 2.9. WB Analysis of Cell Lysates

HK-2 cells cultured in 60-mm culture dishes were also used for WB analysis. GA treatment and AG pretreatment were performed under the same conditions as those described in Section 2.6. After treatment, cells were washed with PBS(-) and lysed in RIPA buffer supplemented with an EDTA-free protease inhibitor cocktail. Cell lysates were collected using centrifugation, and protein concentrations were determined using the BCA protein assay kit with BSA as the standard. Cell lysates (12 μg protein) were mixed with Laemmli sample buffer supplemented with 2-mercaptoethanol (2-ME; 95:5, *v*/*v*) and heated at 95 °C for 5 min. The first lane was loaded with Precision Plus Protein Dual Xtra Standards, a prestained molecular weight marker, whereas the second lane was loaded with Prestained Protein Marker and visualized via chemiluminescent detection. Cell lysates were loaded from the third lane onward. Equal amounts of protein were separated through a 4–15% gradient SDS-polyacrylamide gel electrophoresis and transferred onto PVDF membranes at 40 mA per gel for 1.5 h. Membranes were blocked with 5% skim milk in PBS(-) containing 0.05% Tween-20 (PBS-T) (SM-PBS-T) for 30 min at RT. Primary antibodies were diluted in 0.5% SM-PBS-T and incubated on the blocked membranes under the following conditions: anti-Beclin-1 antibody (1:2000; overnight at 4 °C), anti-ATG7 antibody (1:10,000; 2 h at RT), anti-HSP90α antibody (1:10,000; overnight at 4 °C), anti-HSP90β antibody (1:100,000; 2 h at RT), anti-HSP90 antibody (1:1000; overnight at 4 °C), anti-LC3 antibody (1:5000; 1 h at RT), anti-p62 antibody (ab207305; 1:80,000; 2 h at RT), and anti-β-actin antibody (1:5000; 1 h at RT). After washing with PBS-T, the membranes were incubated with the appropriate HRP-conjugated secondary antibody for 1 h at RT. HRP-linked goat anti-mouse IgG antibody (1:5000) or HRP-linked donkey anti-rabbit IgG antibody (1:5000) was used according to the species of the primary antibody. Immunoreactive bands were visualized using the ImmunoStar LD detection kit. Band intensities were quantified using a Fusion FX imager (software version: 17.03; M&S Instruments, Osaka, Japan) and expressed as arbitrary units (AU). WB experiments and densitometric analyses were not performed under blinded conditions. After imaging, the membranes were stripped using WB stripping solution and reprobed with an anti-β-actin antibody as a loading control.

### 2.10. Statistical Analysis

Statistical analyses were performed using StatFlex (version 6; Artech Co., Ltd., Osaka, Japan). Data are presented as mean ± standard deviation (SD). Differences between two independent animal groups were analyzed using the Mann–Whitney U test. For cell experiments involving multiple treatment conditions, each treatment condition was considered an experimental group and analyzed using one-way analysis of variance, followed by Tukey’s multiple comparison test. For the LC3 analysis in the independent GA treatment experiment, selected pairwise comparisons were additionally evaluated using Bonferroni correction as a supplementary analysis. This supplementary analysis did not replace the primary Tukey analysis. Statistical significance was set at *p*-value < 0.05.

## 3. Results

### 3.1. Histological Analysis and Immunohistochemical Staining of Rat Kidneys

**Figure 1:**
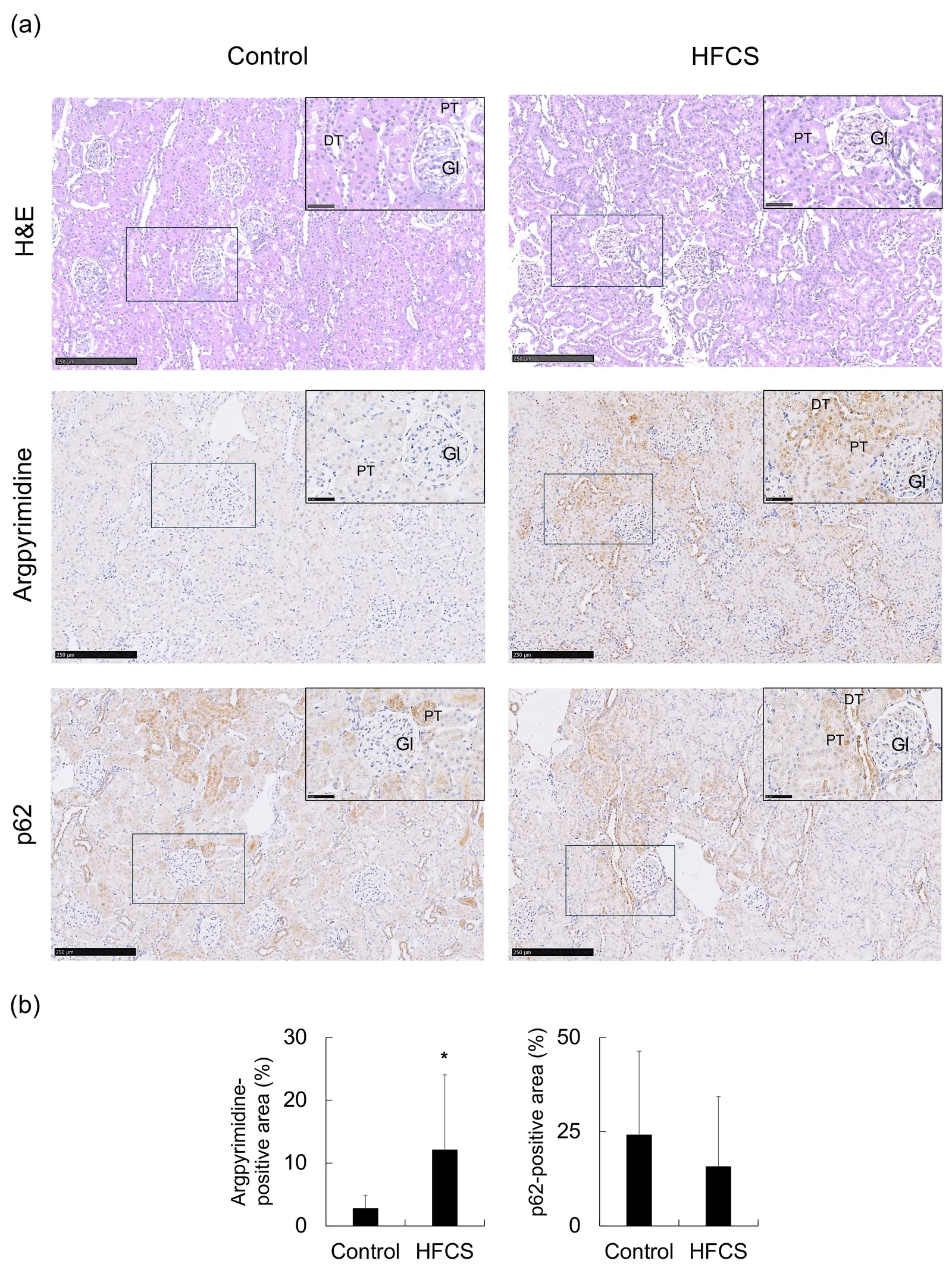
Histological and immunohistochemical analyses of rat kidneys. (**a**) Histological analysis with hematoxylin and eosin (H&E) staining and immunohistochemical staining using antibodies against argpyrimidine and p62. Sample sizes (control/high-fructose corn syrup [HFCS] group) were as follows: H&E, 8/10; argpyrimidine, 9/10; and p62, 9/8. The main images are shown at low magnification (scale bars = 250 μm), with higher-magnification images inset in the upper right corner (scale bars = 50 μm). PT, proximal tubule; DT, distal tubule; Gl, glomerulus. (**b**) Quantification of argpyrimidine- and p62-positive areas in rat kidneys based on immunohistochemical staining. Sample sizes were the same as those in (**a**). Data are shown as mean ± standard deviation (SD). * *p* < 0.05 vs. control (Mann–Whitney U test).

Histological changes in the kidneys were evaluated using H&E staining, and the immunoreactivity of argpyrimidine and p62 was examined via immunohistochemistry. No apparent histological abnormalities were observed in the kidneys of rats receiving HFCS compared with those of the control group (Figure 1a). In contrast, markedly stronger argpyrimidine immunoreactivity was observed in renal tubular epithelial cells of rats receiving HFCS compared with that in the control group, involving both proximal and distal tubular profiles. The immunoreactivity was predominantly granular and cytoplasmic, with occasional nuclear staining. A similar staining pattern was also observed in some glomerular cells, although the staining intensity was mild to moderate. Quantitative analysis confirmed that argpyrimidine-positive areas were higher in the HFCS group than in the control group (mean score: 12.1% vs. 2.8%, *p* < 0.05; Figure 1b). Although p62 immunoreactivity showed a similar staining pattern, it appeared lower in the HFCS group (15.8%) than in the control group (24.1%); however, the difference was not statistically significant.

### 3.2. Effects of GA on NADPH and ATP Levels and Morphological Changes in HK-2 Cells

**Figure 2:**
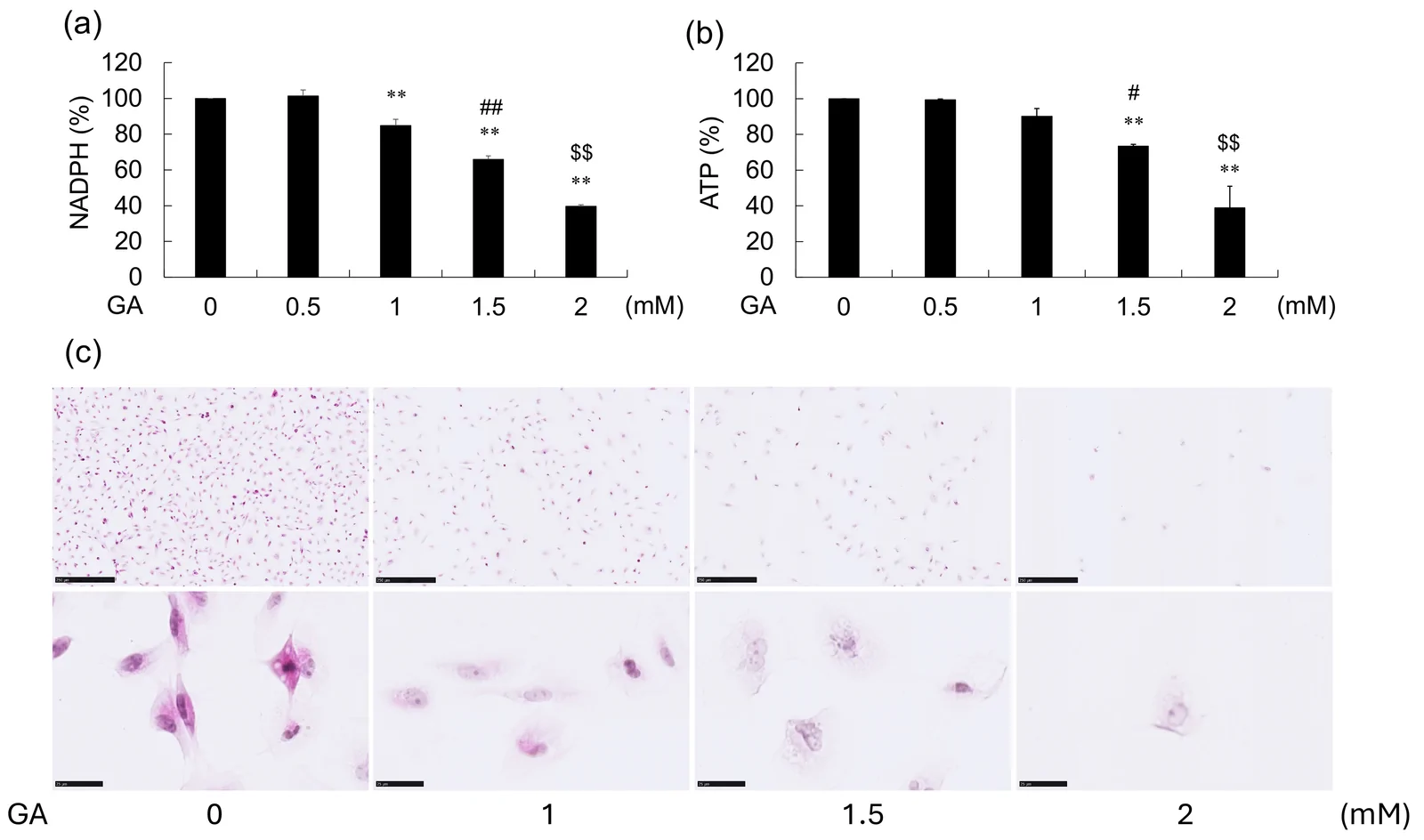
Effects of glyceraldehyde (GA) on nicotinamide adenine dinucleotide phosphate (NADPH) and adenosine triphosphate (ATP) levels and morphological changes in human renal epithelial cells (HK-2). (**a**) NADPH levels were assessed using the Cell Counting Kit-8 (WST-8) assay in three independent experiments. Each of the three experiments included seven wells per condition, and the average value was calculated. (**b**) ATP levels were assessed using the CellTiter-Glo luminescent assay in three independent experiments. Each of the three experiments included five wells per condition, and the average was calculated. (**a**,**b**) Cells were treated with 0, 0.5, 1, 1.5, and 2 mM GA for 24 h. Data are shown as mean ± SD (n = 3). *p*-values were based on Tukey’s test. ** *p* < 0.01 vs. 0 mM GA; # *p* < 0.05 vs. 1 mM GA; ## *p* < 0.01 vs. 1 mM GA; $$ *p* < 0.01 vs. 1.5 mM GA. (**c**) Representative H&E-stained images of HK-2 cells cultured in glass chamber slides following treatment with 0, 1.0, 1.5, and 2.0 mM GA. Four independent experiments were performed, each including all four GA concentrations. Representative images are shown. Upper panels: scale bars = 250 μm. Lower panels: scale bars = 25 μm.

GA treatment induced a concentration-dependent reduction in NADPH and ATP levels in HK-2 cells (Figure 2a,b). NADPH levels decreased to 85%, 66%, and 40% of the control levels following treatment with 1, 1.5, and 2 mM GA, respectively (Figure 2a). Similarly, intracellular ATP levels decreased to 90%, 73%, and 39% after treatment with 1, 1.5, and 2 mM GA, respectively (Figure 2b). Significant reductions in NADPH and ATP levels were observed at 1.5 and 2 mM GA compared with the control (*p* < 0.01). Under a phase-contrast microscope, a small number of cells were found floating in the culture medium at 1.5 and 2 mM GA. At the same concentrations, the dish-attached cells showed changes in their contours (Figure S1). H&E-stained untreated HK-2 cells exhibited a typical epithelial morphology. GA treatment induced concentration-dependent morphological alterations, characterized by a gradual reduction in cell density and progressive cell rounding (Figure 2c).

### 3.3. Effects of GA Treatment on HSP90α Expression in HK-2 Cells

**Figure 3:**
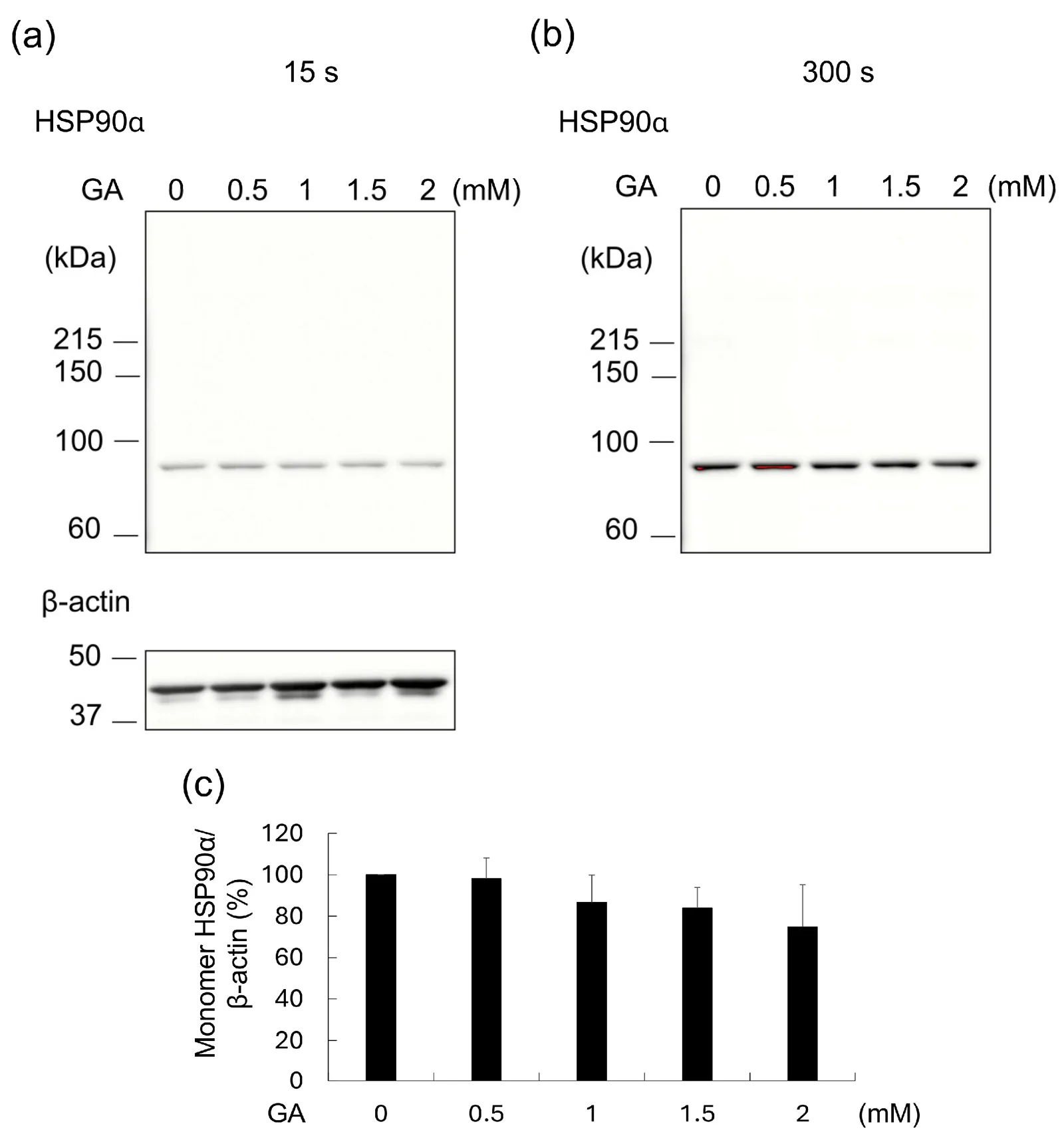
Western blot (WB) analysis of heat shock protein 90 alpha (HSP90α) levels in HK-2 cells treated with GA. Cells were treated with 0, 0.5, 1, 1.5, and 2 mM GA for 24 h. HK-2 cell lysates (12 μg protein/lane) were loaded onto 4–15% polyacrylamide gradient gels. Three independent experiments were performed. (**a**) Proteins on polyvinylidene fluoride (PVDF) membranes were probed with anti-HSP90α and anti-β-actin antibodies. HSP90α was detected with a 15 s exposure. β-actin was used as the loading control. (**b**) The same HSP90α membrane was re-exposed for 300 s. (**c**) Protein levels of HSP90α monomer normalized to β-actin. Data are shown as mean ± SD (n = 3). *p*-values were based on Tukey’s test.

The protein levels of HSP90α were examined through WB analysis. Under both normal and overexposure conditions, only the HSP90α monomer band was detected, whereas no high molecular weight (HMW)-HSP90α bands were observed in any of the experimental groups (Figure 3a,b). Consistent with these findings, quantitative analysis revealed no significant changes in HSP90α protein levels among the treatment groups (Figure 3c).

### 3.4. Effects of GA Treatment on HSP90β Expression in HK-2 Cells

**Figure 4:**
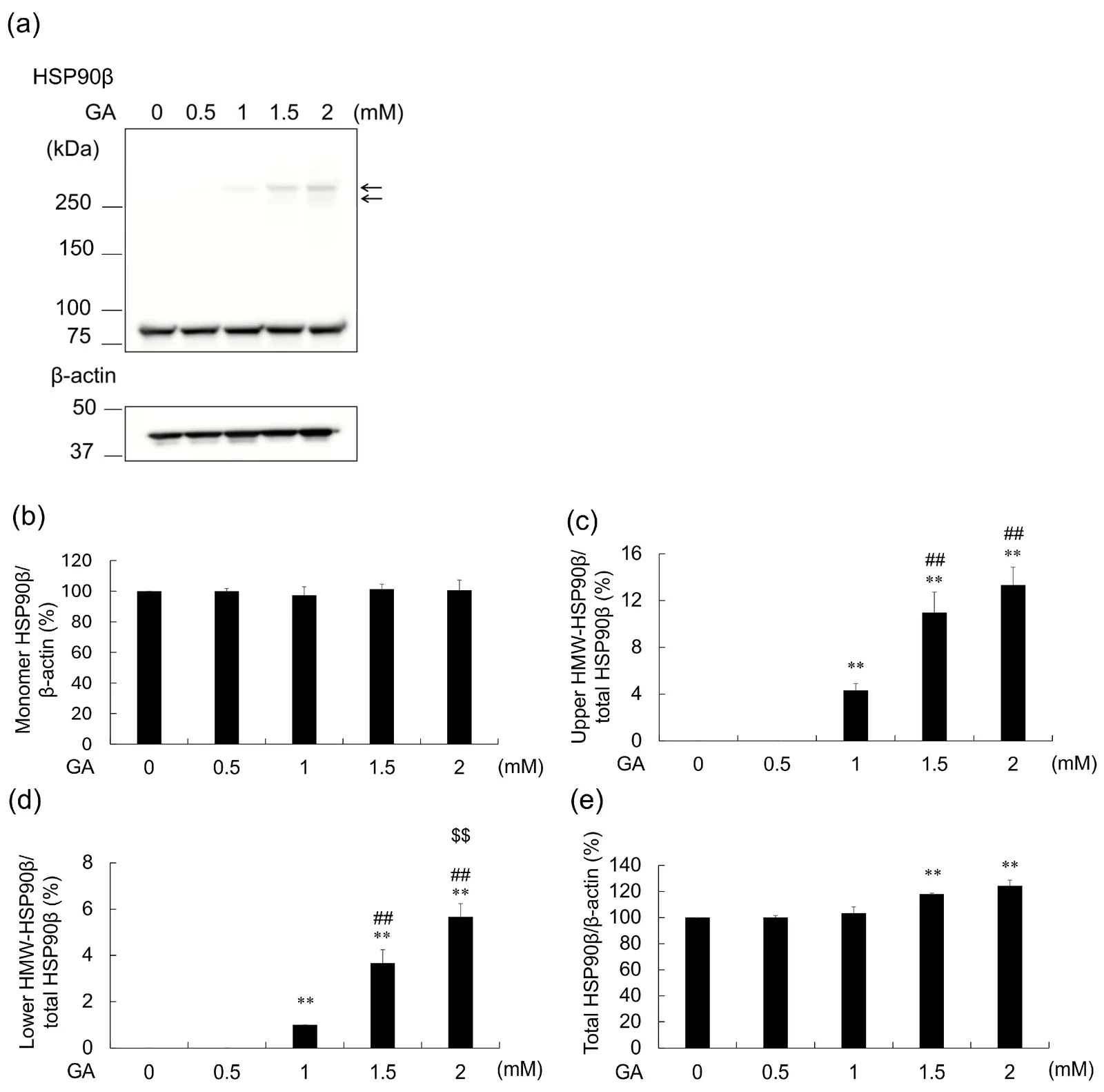
WB analysis of heat shock protein 90 beta (HSP90β) levels in HK-2 cells treated with GA. Cells were treated with 0, 0.5, 1, 1.5, and 2 mM GA for 24 h. HK-2 cell lysates (12 μg protein/lane) were loaded onto 4–15% polyacrylamide gradient gels. Three independent experiments were performed. (**a**) Proteins on PVDF membranes were probed with anti-HSP90β and anti-β-actin antibodies. β-actin was used as the loading control. Upper and lower arrows indicate the upper and lower high molecular weight (HMW)-HSP90β bands, respectively. (**b**) Protein levels of HSP90β monomer normalized to β-actin. (**c**) Protein levels of upper HMW-HSP90β/total HSP90β. (**d**) Protein levels of lower HMW-HSP90β/total HSP90β. (**e**) Protein levels of total HSP90β normalized to β-actin. (**b**–**e**) Data are shown as mean ± SD (n = 3). *p*-values were based on Tukey’s test. ** *p* < 0.01 vs. 0 mM GA; ## *p* < 0.01 vs. 1 mM GA; $$ *p* < 0.01 vs. 1.5 mM GA.

WB analysis was performed to determine HSP90β expression. In untreated HK-2 cells, only the HSP90β monomer band was detected (Figure 4a). Following GA treatment, two additional HMW-HSP90β bands with apparent molecular weights of approximately 270 and 305 kDa gradually appeared. These HMW species were first detectable at 1 mM GA and became progressively more intense at 1.5 and 2 mM GA, whereas the intensity of the monomer band remained largely unchanged. Quantitative analysis confirmed that HSP90β monomer protein levels did not differ significantly among the experimental groups (Figure 4b). In contrast, both the upper and lower HMW-HSP90β/total HSP90β ratios were increased significantly relative to untreated control, reaching 4%, 11%, and 13% for the upper HMW species and 1%, 4%, and 6% for the lower HMW species following treatment with 1, 1.5, and 2 mM GA, respectively (Figure 4c,d). Furthermore, total HSP90β protein levels, normalized to β-actin, were significantly increased to 118% and 124% following treatment with 1.5 and 2 mM GA, respectively, compared with the levels in the untreated control (Figure 4e).

### 3.5. Effects of GA Treatment on Beclin-1 Expression in HK-2 Cells

**Figure 5:**
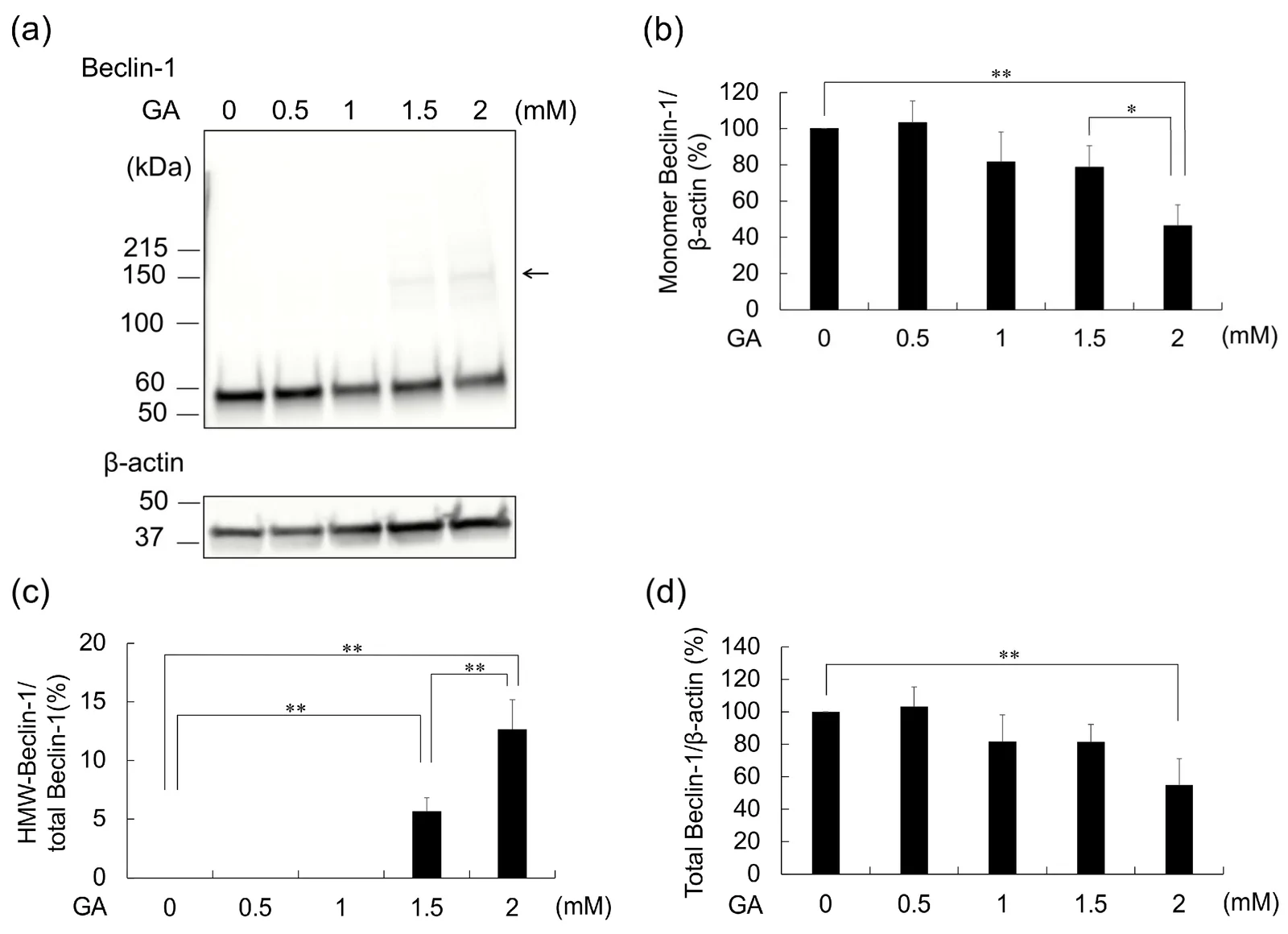
WB analysis of Beclin-1 protein levels in HK-2 cells treated with GA. Cells were treated with 0, 0.5, 1, 1.5, and 2 mM GA for 24 h. HK-2 cell lysates (12 μg protein/lane) were loaded onto 4–15% polyacrylamide gradient gels. Three independent experiments were performed. (**a**) Proteins on PVDF membranes were probed with anti-Beclin-1 and anti-β-actin antibodies. β-actin was used as the loading control. The arrow indicates the HMW-Beclin-1 band. (**b**) Protein levels of Beclin-1 monomer normalized to β-actin. (**c**) Protein levels of HMW-Beclin-1/total Beclin-1. (**d**) Protein levels of total Beclin-1 normalized to β-actin. (**b**–**d**) Data are shown as mean ± SD (n = 3). *p*-values were based on Tukey’s test. * *p* < 0.05; ** *p* < 0.01.

Changes in Beclin-1 protein expression were evaluated through WB analysis. In untreated and low-concentration GA-treated cells, only the Beclin-1 monomer band was observed (Figure 5a). Following treatment with 1.5 and 2 mM GA, a single HMW-Beclin-1 band was detected at approximately 132 kDa. Quantitative analysis revealed that Beclin-1 monomer protein levels, normalized to β-actin, were decreased to 79% and 46% following treatment with 1.5 and 2 mM GA, respectively, compared with the levels in the untreated control (Figure 5b). In contrast, the HMW-Beclin-1/total Beclin-1 ratios were significantly increased to 6% and 13% in the 1.5 and 2 mM GA-treated groups, respectively (Figure 5c). Compared with the levels in the untreated controls, total Beclin-1 protein levels, normalized to β-actin, were significantly decreased to 55% in HK-2 cells treated with 2 mM GA (Figure 5d).

### 3.6. Effects of GA Treatment on ATG7 Expression in HK-2 Cells

**Figure 6:**
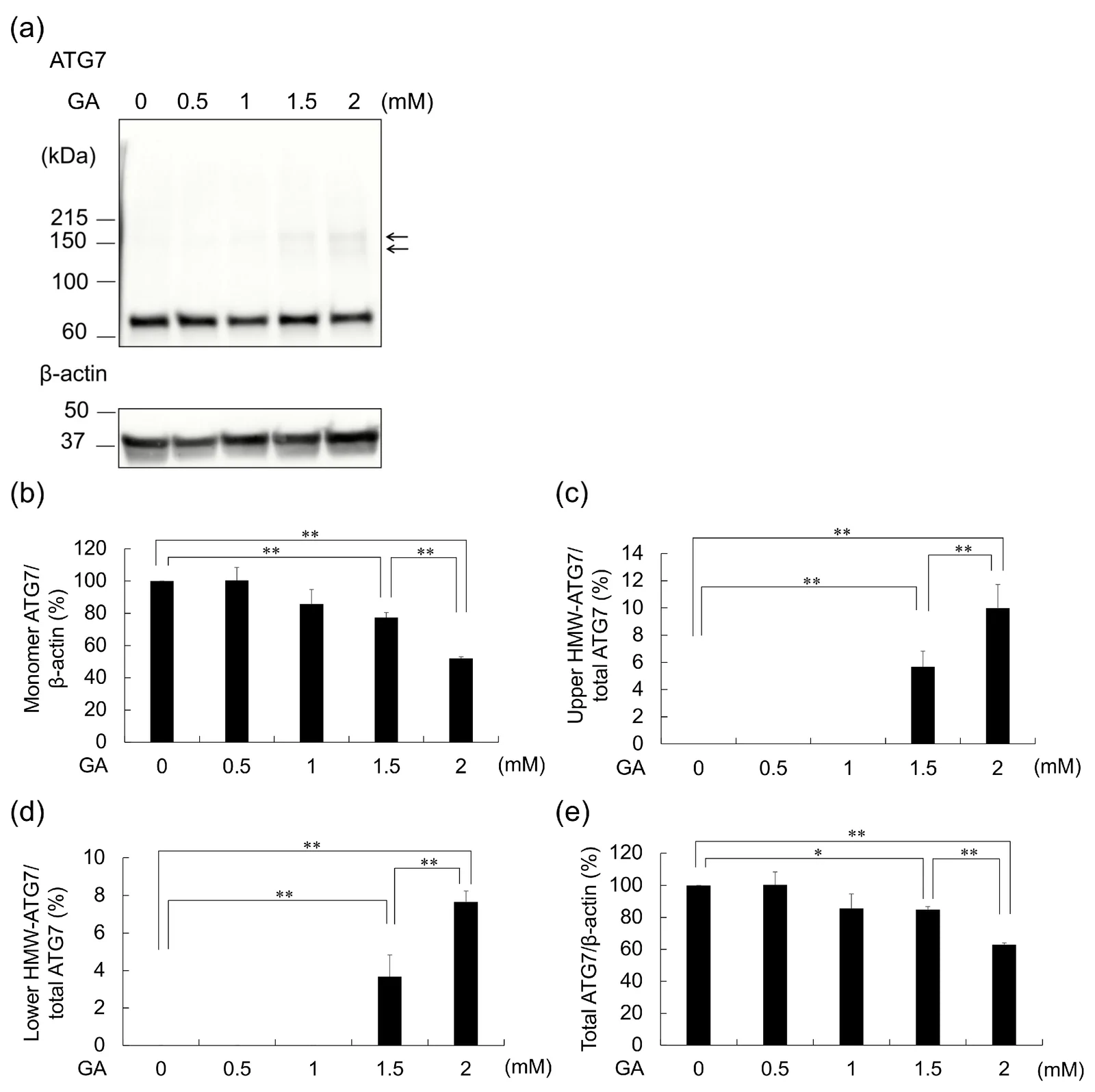
WB analysis of ATG7 protein levels in HK-2 cells treated with GA. Cells were treated with 0, 0.5, 1, 1.5, and 2 mM GA for 24 h. HK-2 cell lysates (12 μg protein/lane) were loaded onto 4–15% polyacrylamide gradient gels. Three independent experiments were performed. (**a**) Proteins on PVDF membranes were probed with anti-ATG7 and anti-β-actin antibodies. β-actin was used as the loading control. Upper and lower arrows indicate the upper and lower HMW-ATG7 bands, respectively. (**b**) Protein levels of ATG7 monomer normalized to β-actin. (**c**) Protein levels of upper HMW-ATG7/total ATG7. (**d**) Protein levels of lower HMW-ATG7/total ATG7. (**e**) Protein levels of total ATG7 normalized to β-actin. (**b**–**e**) Data are shown as mean ± SD (n = 3). *p*-values were based on Tukey’s test. * *p* < 0.05; ** *p* < 0.01.

ATG7 expression was assessed via WB analysis. Under control conditions, only the ATG7 monomer band was detected (Figure 6a). Treatment with 1.5 and 2 mM GA induced the appearance of two additional HMW-ATG7 bands, including an upper band (approximately 168 kDa) and a lower band (approximately 137 kDa). The protein levels of the ATG7 monomer, normalized to β-actin, were significantly decreased to 77% and 52% following treatment with 1.5 and 2 mM GA, respectively, compared with the levels in the untreated control (Figure 6b). In contrast, both the upper and lower HMW-ATG7/total ATG7 ratios were significantly increased in the 1.5 and 2 mM GA-treated groups, reaching 6% and 10% for the upper HMW band and 4% and 8% for the lower HMW band, respectively (Figure 6c,d). Compared with the levels in the untreated control, total ATG7 protein levels, normalized to β-actin, were significantly reduced to 85% and 63% following treatment with 1.5 and 2 mM GA, respectively (Figure 6e).

### 3.7. Effects of GA Treatment on LC3-I, LC3-II, and p62 Expression in HK-2 Cells

**Figure 7:**
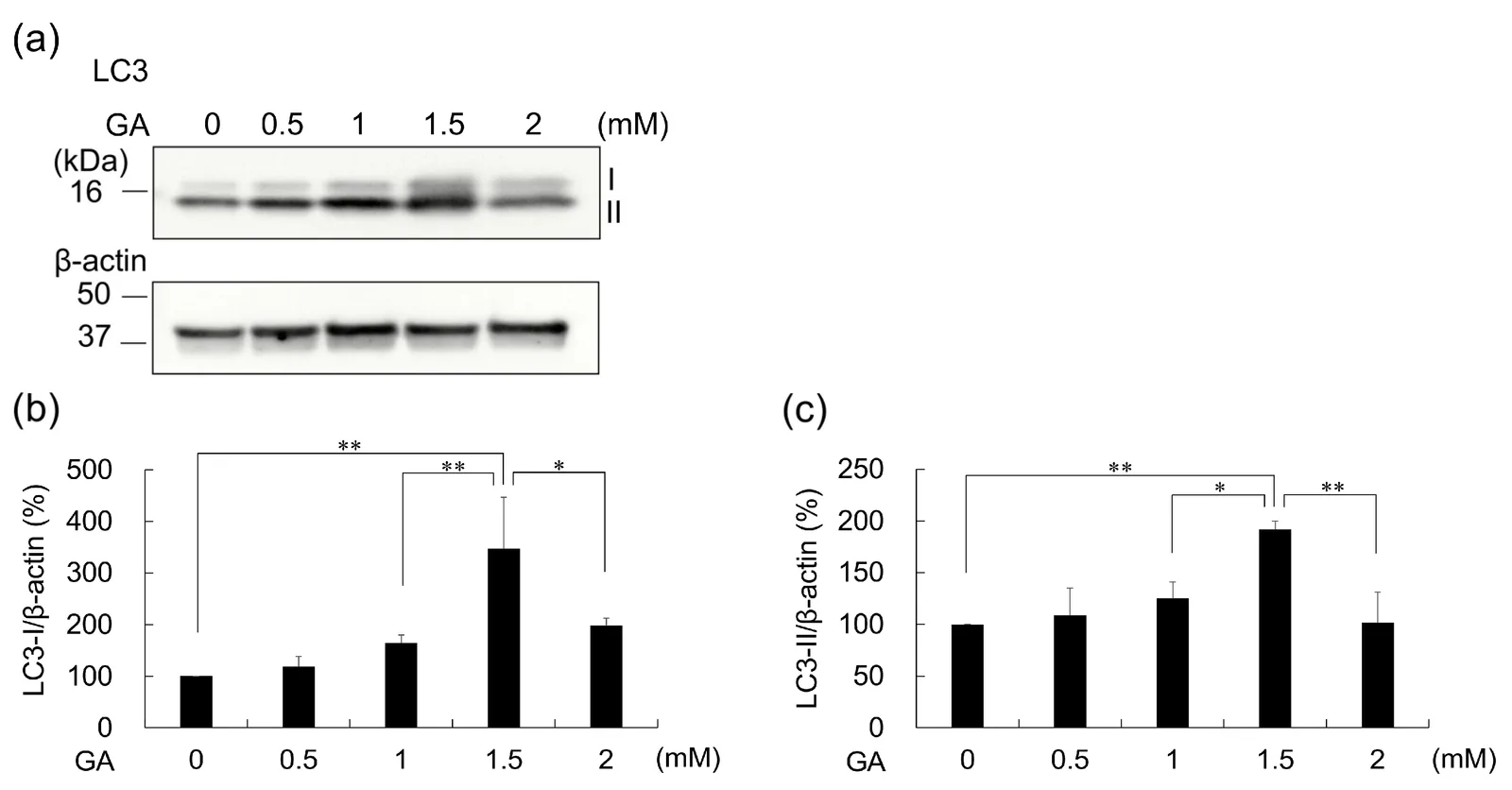
WB analysis of microtubule-associated protein 1 light chain 3 (LC3) protein levels in HK-2 cells treated with GA. Cells were treated with 0, 0.5, 1, 1.5, and 2 mM GA for 24 h. HK-2 cell lysates (12 μg protein/lane) were loaded onto 4–15% polyacrylamide gradient gels. Three independent experiments were performed. (**a**) Proteins on PVDF membranes were probed with anti-LC3 and anti-β-actin antibodies. β-actin was used as the loading control. The positions of LC3-I and LC3-II are indicated by I and II. (**b**) Protein levels of LC3-I normalized to β-actin. (**c**) Protein levels of LC3-II normalized to β-actin. (**b**,**c**) Data are shown as mean ± SD (n = 3). *p*-values were based on Tukey’s test. * *p* < 0.05; ** *p* < 0.01.

**Figure 8:**
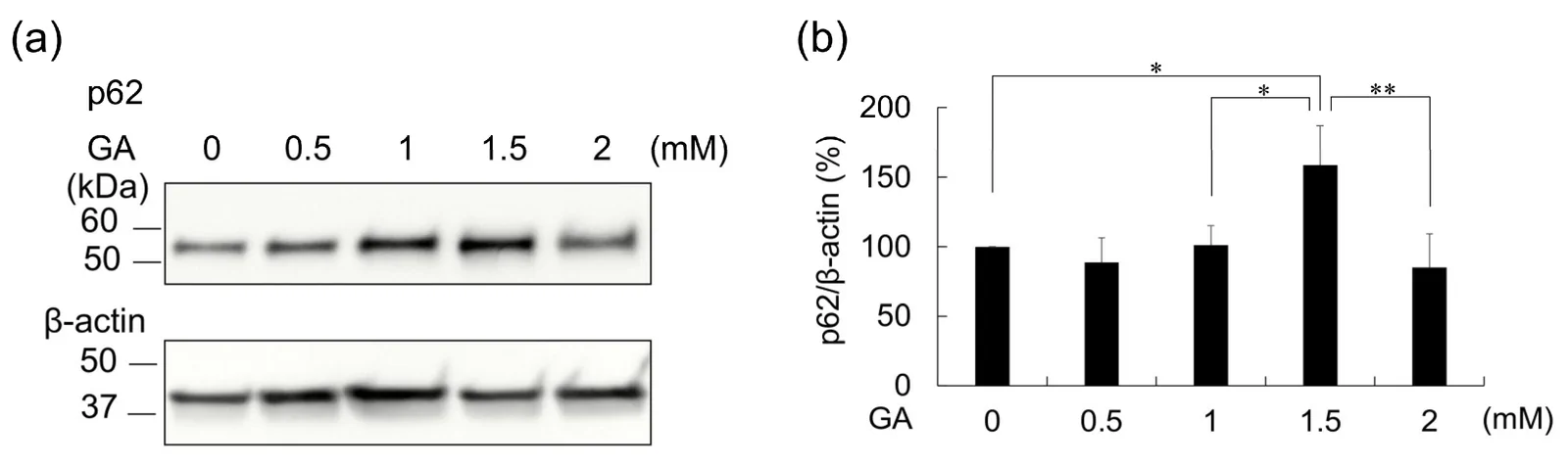
WB analysis of p62 protein levels in HK-2 cells treated with GA. Cells were treated with 0, 0.5, 1, 1.5, and 2 mM GA for 24 h. HK-2 cell lysates (12 μg protein/lane) were loaded onto 4–15% polyacrylamide gradient gels. Three independent experiments were performed. (**a**) Proteins on PVDF membranes were probed with anti-p62 and anti-β-actin antibodies. β-actin was used as the loading control. (**b**) Protein levels of p62 normalized to β-actin. Data are shown as mean ± SD (n = 3). *p*-values were based on Tukey’s test. * *p* < 0.05; ** *p* < 0.01.

LC3-I, LC3-II, and p62 protein levels were analyzed via WB analysis. No HMW forms of LC3-I or LC3-II were detected (Figure 7a). No significant changes in the protein levels of LC3-I or LC3-II were observed in HK-2 cells treated with 0.5 mM GA compared with the levels in the untreated control (Figure 7b,c). Although the protein levels of LC3-I and LC3-II following treatment with 1 mM GA did not show significant differences compared with the levels in the control group, according to Tukey’s test, LC3-I protein levels were increased according to Bonferroni’s test (*p* = 0.01985). In contrast, treatment with 1.5 mM GA markedly increased the protein levels of both LC3-I and LC3-II to 347% and 192%, respectively. This increase was significantly attenuated by treatment with 2 mM GA, resulting in LC3-I and LC3-II levels of 199% and 102%, respectively. Although the upregulation of LC3-I and LC3-II protein levels following treatment with 2 mM GA did not show significant differences compared with the levels in the control group, according to Tukey’s test, LC3-I protein levels increased according to Bonferroni’s test (*p* = 0.00234). Similarly, no significant changes in p62 protein levels were observed following treatment with 0.5 or 1 mM GA (Figure 8). Treatment with 1.5 mM GA markedly increased p62 protein levels to 159%. In contrast, treatment with 2 mM GA significantly attenuated the increase in p62 protein levels to 85%.

### 3.8. Effects of AG Pretreatment on NADPH and ATP Levels and Morphological Changes in GA-Treated HK-2 Cells

**Figure 9:**
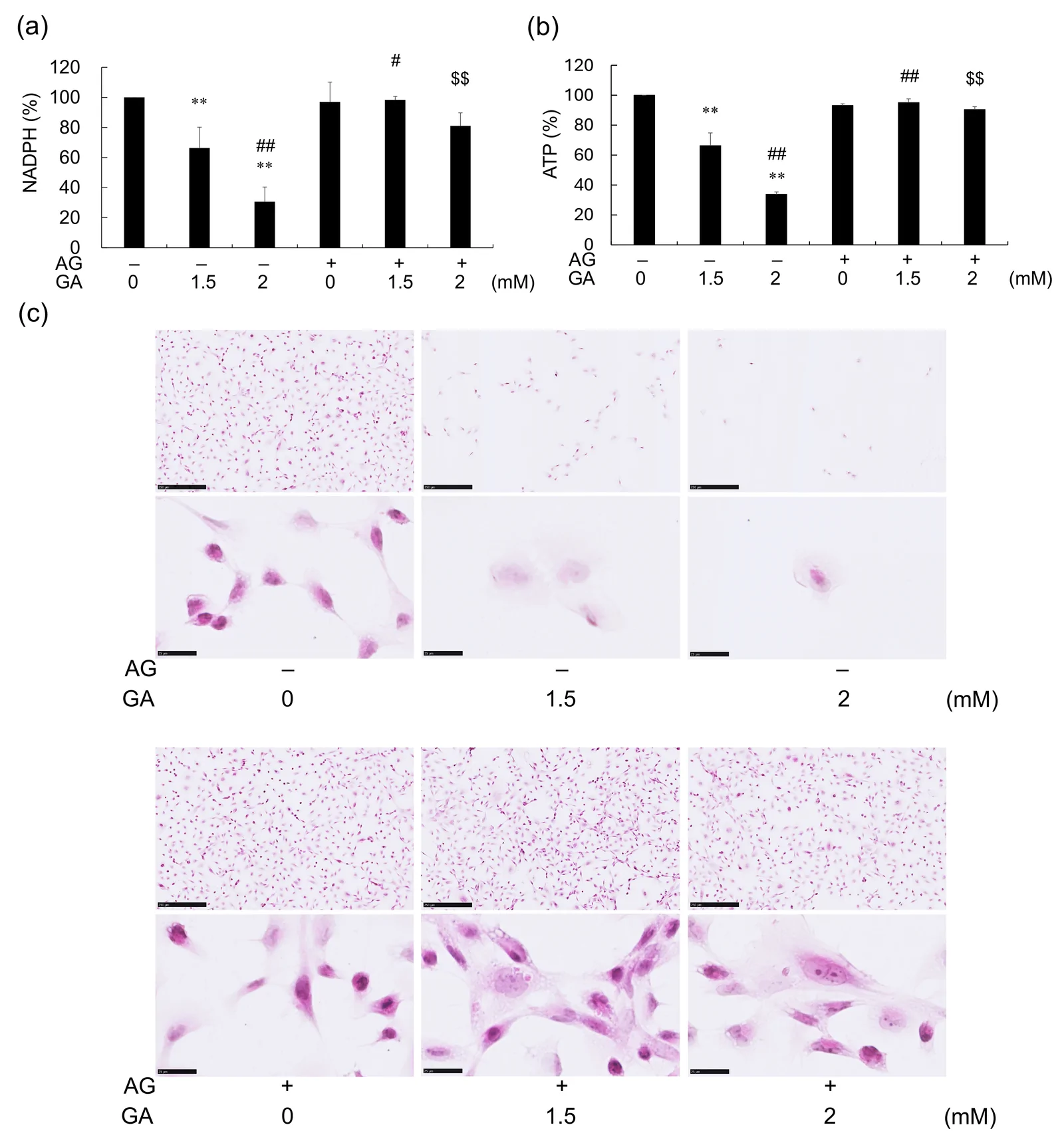
Effects of GA and aminoguanidine (AG) on NADPH and ATP levels and morphological changes in HK-2 cells. (**a**) NADPH levels were assessed using the WST-8 assay in three independent experiments. One experiment was performed using seven wells and the average was calculated. (**b**) ATP levels were assessed using the CellTiter-Glo luminescent assay in three independent experiments. One experiment was performed using five wells and the average was calculated. (**a**,**b**) Cells were treated with 0 or 8 mM AG for 2 h, followed by 0, 1.5, and 2 mM GA for 24 h. Data are shown as mean ± SD (n = 3). *p*-values were based on Tukey’s test. ** *p* < 0.01 vs. 0 mM GA + 0 mM AG; # *p* < 0.05 vs. 1.5 mM GA + 0 mM AG; ## *p* < 0.01 vs. 1.5 mM GA + 0 mM AG; $$ *p* < 0.01 vs. 2 mM GA + 0 mM AG. (**c**) Representative H&E-stained images of HK-2 cells cultured on glass chamber slides following GA treatment in the presence or absence of AG. The first set of images shows cells treated with 0, 1.5, and 2 mM GA without AG pretreatment. The second set shows cells treated with 0, 1.5, and 2 mM GA following AG pretreatment. The experiments were independently repeated four times. Low-magnification images: scale bars = 250 μm. High-magnification images: scale bars = 25 μm.

In the absence of AG pretreatment, NADPH levels decreased to 66% and 31% following treatment with 1.5 and 2 mM GA, respectively (Figure 9a). In contrast, pretreatment with 8 mM AG preserved NADPH levels at 97%, 98%, and 81% following treatment with 0, 1.5, and 2 mM GA, respectively, with no significant differences among the AG-pretreated groups. Likewise, ATP levels decreased to 66% and 34% after treatment with 1.5 and 2 mM GA in the absence of AG pretreatment (Figure 9b), whereas pretreatment with AG maintained ATP levels at 93%, 95%, and 90% following treatment with 0, 1.5, and 2 mM GA, respectively; no significant differences were detected among these groups. H&E-stained HK-2 cells showed relatively preserved morphology in the AG-pretreated groups compared with the corresponding groups without AG pretreatment (Figure 9c). Consistent with these findings, phase-contrast microscopy revealed less pronounced cell rounding and detachment in the AG-pretreated group than in the corresponding groups without AG pretreatment (Figure S2).

### 3.9. Effects of AG Pretreatment on HSP90β Expression in GA-Treated HK-2 Cells

**Figure 10:**
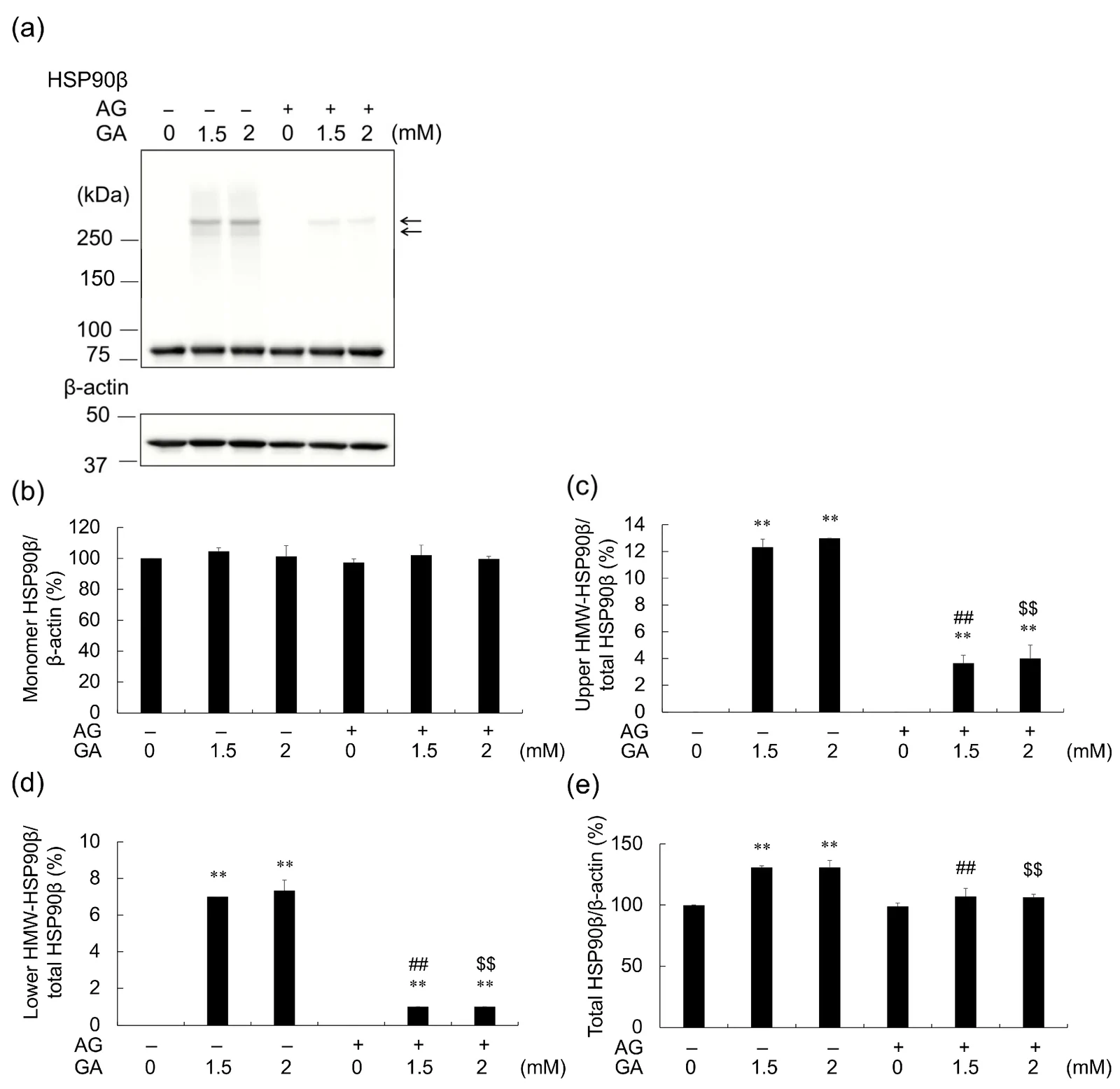
WB analysis of HSP90β levels in HK-2 cells treated with GA and AG. Cells were treated with 0 or 8 mM AG for 2 h, followed by 0, 1.5, and 2 mM GA for 24 h. HK-2 cell lysates (12 μg protein/lane) were loaded onto 4–15% polyacrylamide gradient gels. Three independent experiments were performed. (**a**) Proteins on PVDF membranes were probed with anti-HSP90β and anti-β-actin antibodies. β-actin was used as the loading control. Upper and lower arrows indicate the upper and lower HMW-HSP90β bands, respectively. (**b**) Protein levels of HSP90β monomer normalized to β-actin. (**c**) Protein levels of upper HMW-HSP90β/total HSP90β. (**d**) Protein levels of lower HMW-HSP90β/total HSP90β. (**e**) Protein levels of total HSP90β normalized to β-actin. (**b**–**e**) Data are shown as mean ± SD (n = 3). *p*-values were based on Tukey’s test. ** *p* < 0.01 vs. 0 mM GA + 0 mM AG; ## *p* < 0.01 vs. 1.5 mM GA + 0 mM AG; $$ *p* < 0.01 vs. 2 mM GA + 0 mM AG.

Changes in HSP90β protein levels were evaluated through WB analysis. In the absence of AG, the changes in the HSP90β monomer, HMW species, and total protein levels were consistent with those following GA treatment alone (Figure 10). Pretreatment with 8 mM AG markedly suppressed the GA-induced formation of both the upper and lower HMW-HSP90β bands. Accordingly, AG significantly reduced the upper HMW-HSP90β/total HSP90β ratios from 12% to 4% in HK-2 cells treated with 1.5 mM GA and from 13% to 4% in those treated with 2 mM GA (Figure 10c). Similarly, the lower HMW-HSP90β/total HSP90β ratios were reduced from 7% to 1% following treatment with both concentrations (Figure 10d). AG significantly restored total HSP90β protein levels from 131% to 107% and from 131% to 106% in HK-2 cells treated with 1.5 and 2 mM GA, respectively (Figure 10e).

### 3.10. Effects of AG Pretreatment on Beclin-1 Expression in GA-Treated HK-2 Cells

**Figure 11:**
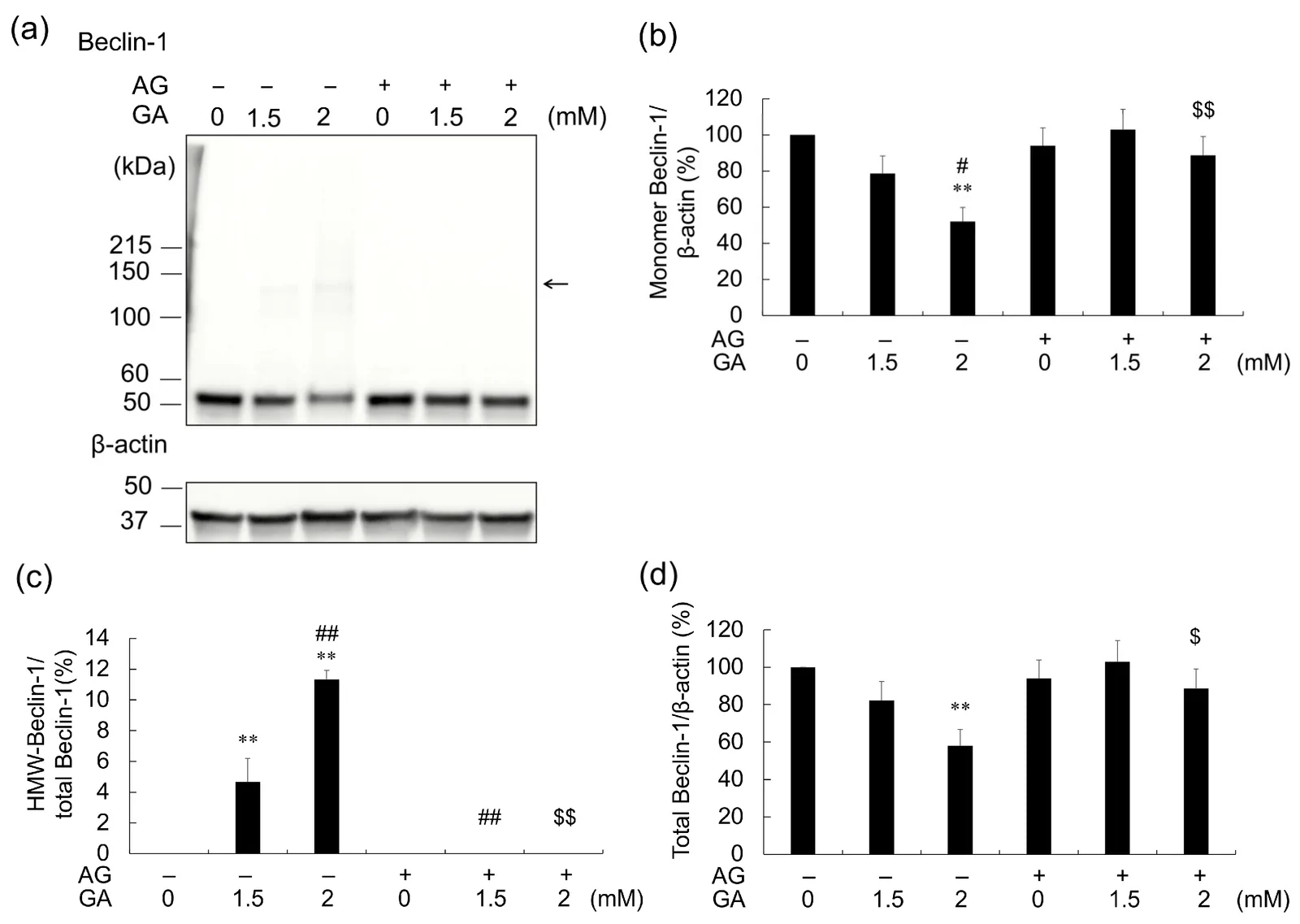
WB analysis of Beclin-1 protein levels in HK-2 cells treated with GA and AG. Cells were treated with 0 or 8 mM AG for 2 h, followed by 0, 1.5, and 2 mM GA for 24 h. HK-2 cell lysates (12 μg protein/lane) were loaded onto 4–15% polyacrylamide gradient gels. Three independent experiments were performed. (**a**) Proteins on PVDF membranes were probed with anti-Beclin-1 and anti-β-actin antibodies. β-actin was used as the loading control. The arrow indicates the HMW-Beclin-1 band. (**b**) Protein levels of Beclin-1 monomer normalized to β-actin. (**c**) Protein levels of HMW-Beclin-1/total Beclin-1. (**d**) Protein levels of total Beclin-1 normalized to β-actin. (**b**–**d**) Data are shown as mean ± SD (n = 3). *p*-values were based on Tukey’s test. ** *p* < 0.01 vs. 0 mM GA + 0 mM AG; # *p* < 0.05 vs. 1.5 mM GA + 0 mM AG; ## *p* < 0.01 vs. 1.5 mM GA + 0 mM AG; $ *p* < 0.05 vs. 2 mM GA + 0 mM AG; $$ *p* < 0.01 vs. 2 mM GA + 0 mM AG.

The protein levels of Beclin-1 were examined through WB analysis. In the absence of AG, the changes in Beclin-1 protein levels were consistent with those following GA treatment alone (Figure 11). Pretreatment with 8 mM AG markedly inhibited the GA-induced formation of the HMW-Beclin-1 band from 5% to 0% in HK-2 cells treated with 1.5 mM GA and from 11% to 0% in those treated with 2 mM GA (Figure 11c). In addition, AG significantly restored Beclin-1 monomer protein levels from 79% to 103% and from 52% to 89% with 1.5 and 2 mM GA, respectively, and increased total Beclin-1 protein levels from 58% to 89% with 2 mM GA (Figure 11b,d).

### 3.11. Effects of AG Pretreatment on ATG7 Expression in GA-Treated HK-2 Cells

**Figure 12:**
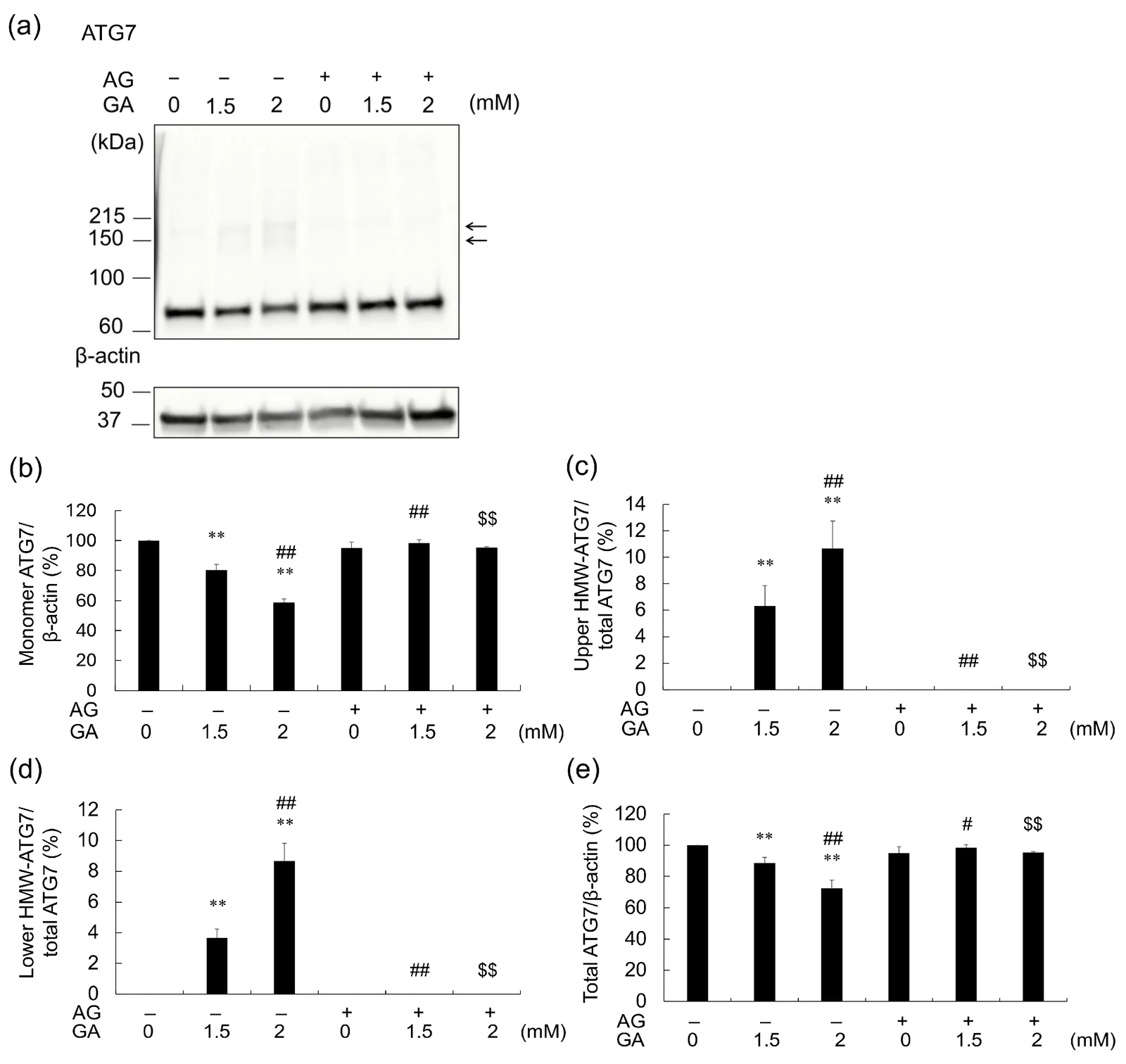
WB analysis of ATG7 protein levels in HK-2 cells treated with GA and AG. Cells were treated with 0 or 8 mM AG for 2 h, followed by 0, 1.5, and 2 mM GA for 24 h. HK-2 cell lysates (12 μg protein/lane) were loaded onto 4–15% polyacrylamide gradient gels. Three independent experiments were performed. (**a**) Proteins on PVDF membranes were probed with anti-ATG7 and anti-β-actin antibodies. β-actin was used as the loading control. Upper and lower arrows indicate the upper and lower HMW-ATG7 bands, respectively. (**b**) Protein levels of ATG7 monomer normalized to β-actin. (**c**) Protein levels of upper HMW-ATG7/total ATG7. (**d**) Protein levels of lower HMW-ATG7/total ATG7. (**e**) Protein levels of total ATG7 normalized to β-actin. (**b**–**e**) Data are shown as mean ± SD (n = 3). *p*-values were based on Tukey’s test. ** *p* < 0.01 vs. 0 mM GA + 0 mM AG; # *p* < 0.05 vs. 1.5 mM GA + 0 mM AG; ## *p* < 0.01 vs. 1.5 mM GA + 0 mM AG; $$ *p* < 0.01 vs. 2 mM GA + 0 mM AG.

ATG7 expression was assessed through WB analysis. In the absence of AG, the changes in ATG7 protein levels were similar to those observed in HK-2 cells treated with GA alone (Figure 12). Pretreatment with 8 mM AG markedly suppressed the formation of both HMW-ATG7 bands, reducing the upper HMW band from 6% to 0% and from 11% to 0%, and the lower HMW band from 4% to 0% and from 9% to 0% in HK-2 cells treated with 1.5 and 2 mM GA, respectively (Figure 12c,d). Furthermore, AG significantly restored ATG7 monomer protein levels from 80% to 98% and from 59% to 95%, and increased total ATG7 protein levels from 89% to 98% and from 73% to 95% in HK-2 cells treated with 1.5 and 2 mM GA, respectively (Figure 12b,e).

### 3.12. Effects of AG Pretreatment on LC3-I, LC3-II, and p62 Expression in GA-Treated HK-2 Cells

**Figure 13:**
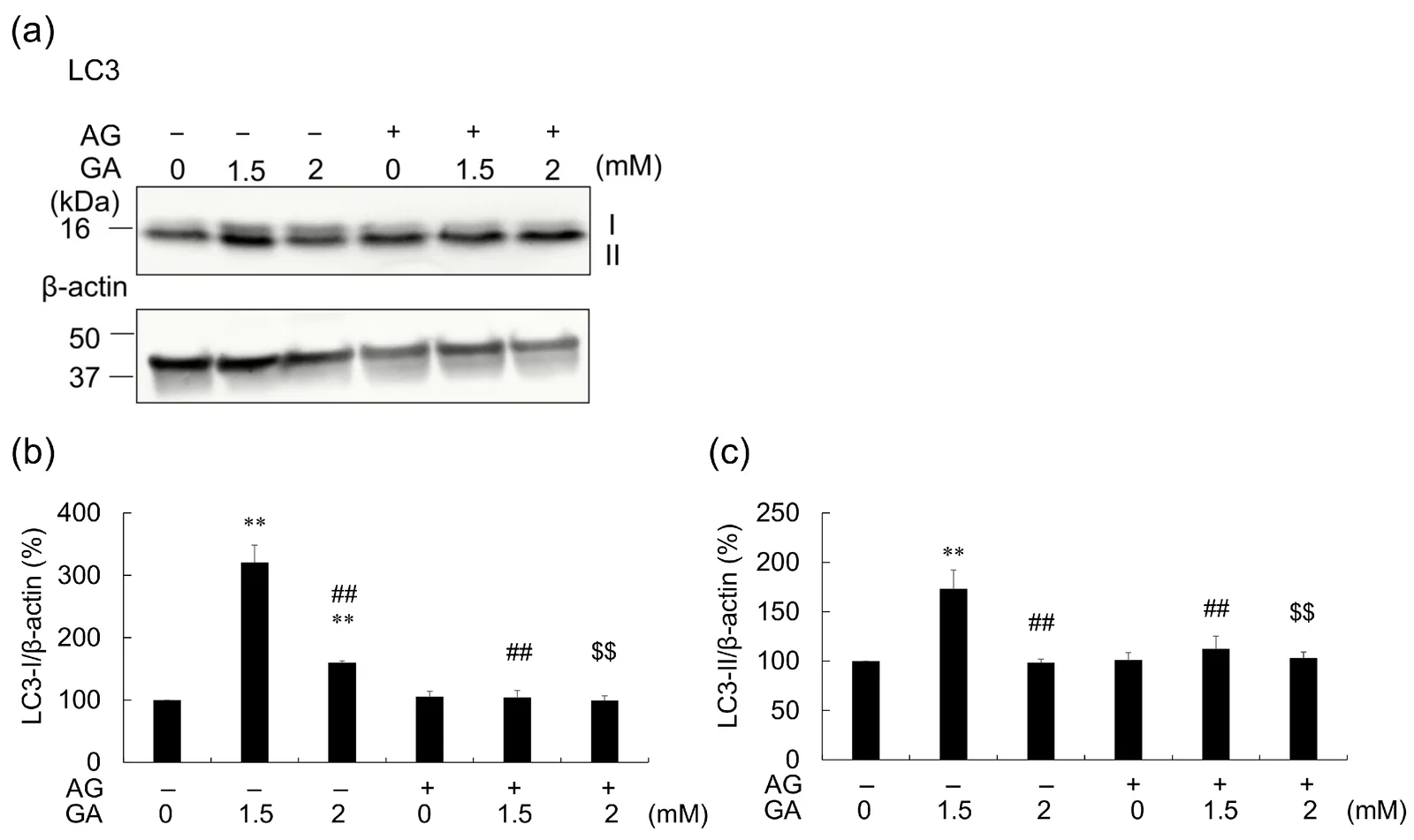
WB analysis of LC3 protein levels in HK-2 cells treated with GA and AG. Cells were treated with 0 or 8 mM AG for 2 h, followed by 0, 1.5, and 2 mM GA for 24 h. HK-2 cell lysates (12 μg protein/lane) were loaded onto 4–15% polyacrylamide gradient gels. Three independent experiments were performed. (**a**) Proteins on PVDF membranes were probed with anti-LC3 and anti-β-actin antibodies. β-actin was used as the loading control. The positions of LC3-I and LC3-II are indicated by I and II. (**b**) Protein levels of LC3-I normalized to β-actin. (**c**) Protein levels of LC3-II normalized to β-actin. (**b**,**c**) Data are shown as mean ± SD (n = 3). *p*-values were based on Tukey’s test. ** *p* < 0.01 vs. 0 mM GA + 0 mM AG; ## *p* < 0.01 vs. 1.5 mM GA + 0 mM AG; $$ *p* < 0.01 vs. 2 mM GA + 0 mM AG.

**Figure 14:**
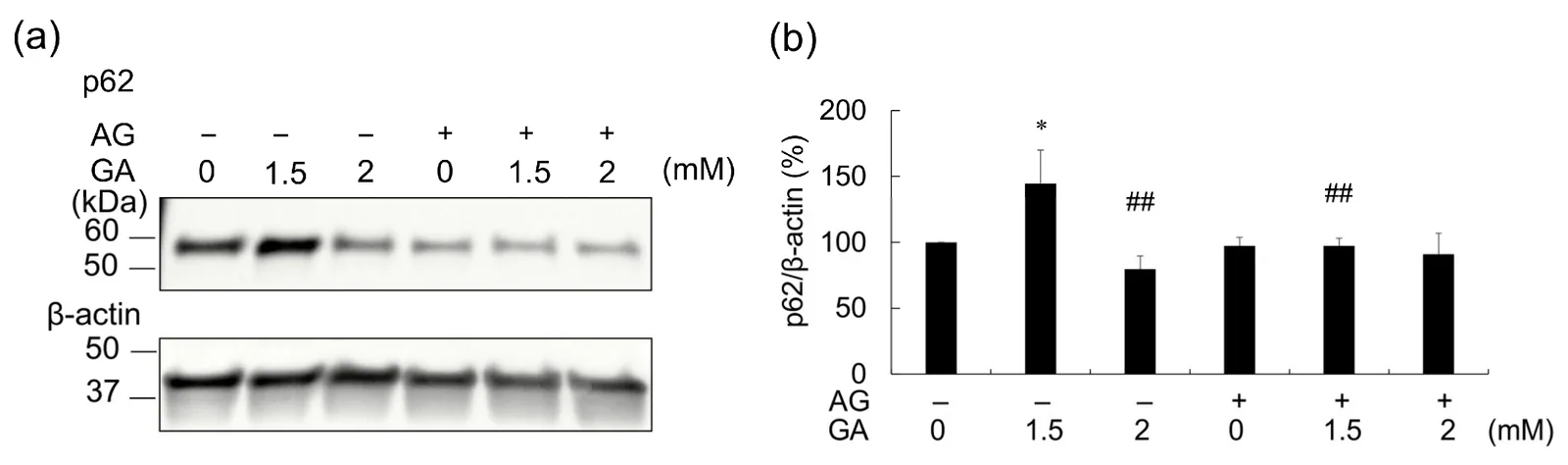
WB analysis of p62 protein levels in HK-2 cells treated with GA and AG. Cells were treated with 0 or 8 mM AG for 2 h, followed by 0, 1.5, and 2 mM GA for 24 h. HK-2 cell lysates (12 μg protein/lane) were loaded onto 4–15% polyacrylamide gradient gels. Three independent experiments were performed. (**a**) Proteins on PVDF membranes were probed with anti-p62 and anti-β-actin antibodies. β-actin was used as the loading control. (**b**) Protein levels of p62 normalized to β-actin. Data are shown as mean ± SD (n = 3). *p*-values were based on Tukey’s test. * *p* < 0.05 vs. 0 mM GA + 0 mM AG; ## *p* < 0.01 vs. 1.5 mM GA + 0 mM AG.

WB analysis was performed for LC3-I, LC3-II, and p62. Treatment with 1.5 mM GA in the absence of AG markedly increased LC3-I and LC3-II protein levels to 321% and 173%, respectively (Figure 13). This increase was significantly attenuated by treatment with 2 mM GA, with LC3-I and LC3-II protein levels of 160% and 98%, respectively. These findings are consistent with those obtained in the corresponding experiments using GA alone. Importantly, AG pretreatment reduced LC3-I and LC3-II protein levels to near-baseline levels. Consistent with the results obtained in the GA-only experiments, treatment with 1.5 mM GA markedly increased p62 protein levels to 145% (Figure 14). The levels of p62 were lower at 2 mM GA, reaching 80%. Similarly, AG pretreatment restored p62 protein levels to near-baseline levels.

## 4. Discussion

Previous studies have investigated intracellular AGE accumulation by immunohistochemistry in various tissues such as the liver, pancreas, heart, skeletal muscle, and kidney [15,27]. Takahashi et al. demonstrated CML accumulation in PTE cells of streptozotocin-induced DN (STZ-DN) mice [15]. However, to our knowledge, no study has assessed the accumulation of intracellular AGEs in the kidney of healthy animal models. Although we previously demonstrated that the toxic AGE (TAGE) accumulated in the liver and circulation of healthy Wistar rats after 8 weeks of HFCS intake, renal accumulation could not be confirmed [28]. Therefore, in the present study, we examined kidney tissues from the same animals using an anti-argpyrimidine antibody. We found that HFCS intake significantly increased renal argpyrimidine immunoreactivity, particularly in the granular cytoplasm of renal tubular epithelial cells, whereas no apparent morphological abnormalities were observed in H&E-stained sections. In our previous study, rats receiving HFCS showed modest increases in triglycerides, hemoglobin A1c, and blood urea nitrogen, while blood glucose, creatinine, and uric acid levels did not differ significantly between the groups [28]. Although insufficient to induce overt kidney injury, these metabolic changes may have promoted intracellular AGE formation. Argpyrimidine is generated from GA and MG, both of which are produced during glucose and fructose metabolism as well as non-enzymatic glycation reactions [10,11,12,13,27]. As other GA- and/or MG-derived AGEs, including MG-H1 and glyceraldehyde-related pyridinium (GLAP), are likely formed simultaneously, multiple intracellular AGEs may accumulate in the kidney following excessive glucose/fructose intake. In contrast, p62 protein levels, an autophagy-related marker, did not differ significantly between the HFCS and the control groups. This finding contrasts with DN models, wherein p62 accumulation has been reported [29], and suggests that autophagic homeostasis was largely preserved at this early stage of AGE accumulation, consistent with the absence of morphological abnormalities. Interestingly, Takahashi et al. reported that *ATG5* knockdown enhanced CML accumulation in PTE cells of STZ-DN mice [15], suggesting that autophagy contributes to intracellular AGE clearance. Therefore, under the present conditions, basal autophagic activity may still have been sufficient to maintain cellular homeostasis despite increased AGE accumulation. The in vivo model provided evidence of increased renal argpyrimidine accumulation following HFCS intake without overt histological injury, whereas the HK-2 experiments provided a complementary model for examining cellular responses to controlled GA treatment rather than directly reproducing the in vivo phenotype. Thus, these findings should be interpreted as complementary evidence rather than as evidence of a direct mechanistic link between HFCS intake to intracellular GA formation and the HMW protein species observed in HK-2 cells.

The GA concentrations used in this study were selected based on previous reports and our own findings, which demonstrate biological effects of GA at concentrations up to 2 mM and intracellular GA-AGE formation following 2 mM GA [30,31,32]. GA treatment decreased NADPH and ATP levels in a concentration-dependent manner, and these reductions were attenuated by AG, an inhibitor of GA-AGE formation. Similar findings have been reported previously, supporting the notion that GA treatment may disrupt cellular homeostasis [32,33]. Consistent with these metabolic changes, GA also induced concentration-dependent morphological alterations, which were attenuated by AG pretreatment, as observed by phase-contrast microscopy and H&E staining. Although the number of cells appeared to decrease in H&E-stained images with increasing GA concentrations, most cells remained attached during culture, suggesting that the apparent reduction was mainly due to cell detachment during the staining procedure.

The AG concentration used in the present study was selected based on a previous study investigating intracellular toxic AGEs (TAGE), in which 8 mM AG pretreatment for 2 h effectively inhibited glyceraldehyde-induced TAGE formation and loss of cell viability [34]. Based on these findings, 8 mM AG was employed in the present study. Notably, 8 mM AG alone did not significantly alter NADPH or ATP levels under the present experimental conditions.

We focused on HSP90 as a molecular chaperone essential for protein quality control and autophagy, thereby maintaining cellular homeostasis [26,35]. HSP90 comprises two major isoforms, HSP90α and HSP90β, which share approximately 85% sequence identity but differ in their expression patterns and client proteins [36,37]. Under physiological conditions, HSP90 functions as a non-covalent dimer stabilized primarily by hydrophobic interactions [38,39,40,41]. In addition to its molecular chaperone function, HSP90 interacts with multiple autophagy-related proteins and contributes to the regulation of autophagy [42,43,44,45,46]. Although monomeric HSP90α and HSP90β levels were unchanged following GA treatment, the abundance of two HMW-HSP90 species increased in a GA concentration-dependent manner. As the HMW-HSP90β bands were preserved under reducing and denaturing conditions, these species are unlikely to represent native HSP90 dimers and may instead involve covalent cross-linking. Instead, these bands may correspond to complexes between HSP90β and other proteins. Another possibility is that oxidative stress induced by GA may promote covalent protein cross-linking, such as dityrosine formation, which could contribute to the generation of HMW-HSP90β and other HMW immunoreactive species [47,48,49]. However, this possibility remains speculative because dityrosine formation and the specific cross-linking sites were not directly examined in the present study. Ubiquitination or other post-translational modifications may also contribute to the formation of these HMW species; however, this possibility remains speculative, as ubiquitination can produce heterogeneous HMW species that may appear as multiple bands, a ladder, or a smear on WB [50,51], whereas the present study showed discrete HMW bands. Although we previously detected a 173-kDa HMW-HSP90β species in pancreatic cancer cells under the same experimental conditions [52], the larger complexes observed in HK-2 cells suggest that HSP90β may interact with different proteins depending on the cellular environment. Interestingly, monomeric HSP90β levels remained unchanged, whereas total HSP90β increased following treatment with 1.5 and 2 mM GA. As inhibition of HSP90 dimerization has been reported to impair HSP90 function [38], the increase in total HSP90β may represent a compensatory response to maintain protein quality control and cellular homeostasis in the presence of dysfunctional HMW-HSP90β. Notably, HMW-HSP90β was already detectable at 1 mM GA, at which NADPH levels showed a statistically significant reduction, whereas ATP levels did not differ significantly from the control. Thus, HMW-HSP90β formation was not restricted to the concentrations associated with the most pronounced metabolic impairment observed at higher GA concentrations. Nevertheless, a contribution of GA-induced cellular stress to the observed HMW-HSP90β formation cannot be completely excluded. In contrast, no HMW-HSP90α bands were detected, even after prolonged exposure. This difference may reflect the distinct amino acid sequences and client protein profiles of HSP90α and HSP90β [36,53], suggesting that GA-associated HMW formation may preferentially involve HSP90β-containing protein complexes. Furthermore, HMW-HSP90β was not detected using an anti-HSP90 antibody that recognizes both isoforms (Figure S3), possibly because GA-derived modification altered the conformation or masked the antibody epitope. Identification of the cross-linking sites will require future structural analyses. Importantly, AG pretreatment markedly suppressed the appearance of HMW-HSP90β, supporting the involvement of GA-induced AGE-related processes in the generation of these HMW species.

Beclin-1 is a key regulator of autophagosome formation during the early stage of autophagy, and its stability is maintained by HSP90 [22,54]. Altered Beclin-1 expression has been reported under both autophagy induction and inhibition, including in models of kidney injury [55,56,57,58,59]. In the present study, 2 mM GA significantly reduced monomeric Beclin-1, while HMW-Beclin-1 species were detected at 1.5 and 2 mM GA, consistent with GA-associated HMW species. Because HMW-Beclin-1 accounted for only approximately 6% of total Beclin-1 at 1.5 mM GA, its effect on Beclin-1 function was likely limited. In contrast, at 2 mM GA, both monomeric and total Beclin-1 levels were significantly decreased. These changes were attenuated by AG pretreatment, supporting their association with GA-induced AGE-related processes. Previous studies have shown that high-glucose exposure generally decreases Beclin-1 expression in HK-2 cells, although transient increases have also been reported depending on the exposure duration [60,61,62]. As Takahashi et al. reported, CML-modified proteins increased in PTE cells incubated in high-glucose medium [15], intracellular AGEs might be generated and accumulated, thereby contributing to the reduction of Beclin-1 protein levels.

ATG7, like Beclin-1, is involved in the early stage of autophagy and catalyzes the lipidation of LC3-I to generate LC3-II [63,64,65,66,67]. In the present study, GA treatment altered ATG7 protein patterns. Monomeric ATG7 levels decreased following 1.5 and 2 mM GA treatment, accompanied by the concentration-dependent appearance of two HMW-ATG7 species (137 and 168 kDa). As the total ATG7 level was significantly reduced following 1.5 and 2 mM GA treatment, the efficiency of LC3-I lipidation to LC3-II may be reduced, even if HMW-ATG7 retained its activity. ATG7 has also been reported as a client protein regulated by HSP90-mediated quality control and therefore may associate with HSP90 through hydrophobic interactions [40,41,43]. This raises the possibility that GA-induced HMW-HSP90β formation may influence ATG7 stability or function. Although the reduction of ATG7 suggests suppression of early-stage autophagy, this notion is not fully consistent with the changes in LC3-I and LC3-II, indicating that autophagy regulation is more complex.

LC3-I, LC3-II, and p62 are widely used markers for monitoring autophagy [58,59,60,68,69]. However, because LC3-II and p62 are degraded following autophagosome–lysosome fusion, their expression levels alone do not necessarily reflect autophagic activity, particularly when autophagy is impaired at the late stage or excessively activated [70,71,72,73]. In our study, GA treatment at 1.5 mM significantly increased the levels of all three proteins, and this effect was attenuated by AG pretreatment, supporting the involvement of GA-induced AGE-related processes. Concurrent increases in LC3-II and p62 have been reported previously, including in colon cancer cells and cisplatin-treated HK-2 cells [71,72]. Li et al. demonstrated that cisplatin increased LC3-II and p62 levels together with autophagosome accumulation but without an increase in autolysosome formation, indicating impaired late-stage autophagy [72]. Although we did not quantify the numbers of autophagosomes or autolysosomes, the increases in LC3-II and p62 observed at 1.5 mM GA could be compatible with impaired degradation during the late stage of autophagy, although this interpretation cannot be confirmed without direct autophagic flux analysis. In contrast, the reduction in ATG7 levels together with the increase in LC3-I suggests that GA may also affect the early stage of autophagy. Thus, GA may affect proteins and processes associated with different stages of autophagy; however, the direction of autophagic flux cannot be determined from the present data. Recent studies have also implicated HSP90 in the regulation of LC3 and p62 [38,74]. In our study, monomeric HSP90α and HSP90β levels were unchanged after GA treatment, whereas HMW-HSP90β increased in a GA concentration-dependent manner. Therefore, GA-induced HMW-HSP90β formation, together with alterations in Beclin-1 and ATG7, may contribute to the observed changes in LC3 and p62.

Taken together, the changes observed at 1.5 and 2 mM GA, including alterations in Beclin-1, ATG7, LC3, and p62, together with reductions in NADPH and ATP levels and the accompanying morphological changes, suggest that normal cellular homeostasis was not fully maintained under these conditions. Because molecular chaperone- and autophagy-related proteins are involved in protein quality control and cellular stress responses, the simultaneous alterations in these proteins may reflect a broader disturbance of cellular homeostasis rather than an isolated defect in a single autophagy pathway. However, the present data do not establish the precise mechanisms responsible for this disturbance. Therefore, the protein changes observed at 1.5–2 mM GA may involve both AGE-related processes and cellular stress, and their relative contributions cannot be determined.

In this study, we could not identify the specific GA-AGE structures, including argpyrimidine, MG-H1, and GLAP, associated with the observed HMW species of HSP90β and autophagy-related proteins. As HMW species were detected by WB analysis without using anti-AGE or anti-ubiquitin antibodies, we could not directly confirm that these HMW forms were AGE-modified, ubiquitinated, or other post-translationally modified proteins. Moreover, autophagic flux was not directly assessed; therefore, whether GA treatment alters autophagic flux and whether AG alleviates this effect remain unclear [70]. Further studies using lysosomal inhibitors such as bafilomycin A1 are warranted [75]. Although 8 mM AG alone did not significantly alter the measured endpoints, potential effects of AG not captured by the present assays cannot be completely excluded.

## 5. Conclusions

We found that HFCS intake was associated with increased argpyrimidine immunoreactivity in the kidneys of healthy Wistar rats, particularly in renal tubular epithelial cells, suggesting that GA- and/or MG-derived intracellular AGEs may accumulate before overt renal injury develops. In HK-2 cells, GA treatment was associated with reduced NADPH and ATP levels, induced morphological alterations, and the appearance of HMW species of HSP90β, Beclin-1, and ATG7, accompanied by reductions in monomeric and total Beclin-1 and ATG7 levels. AG attenuated these GA-induced alterations, supporting the involvement of GA-induced AGE-related processes. Overall, GA may induce the generation of HMW species of HSP90β and autophagy-related proteins and may contribute to altered cellular homeostasis, with AGE-related processes potentially contributing to these effects.

## 6. Limitation

Following HFCS intake, rats were exposed to glucose and fructose. Although GA may be generated from these sugars, the amount generated remains unclear. It may have been lower than the amount added to HK-2 cells in the in vitro experiments, but this issue was not resolved in the present study. Moreover, HK-2 cells are of human renal origin and thus are species-distinct from rats. The rate of intracellular GA generation and the tolerance for GA may differ between these systems, but this was not investigated in the present study. In the in vitro experiments, GA was added to the culture medium at concentrations of up to 2 mM to establish conditions in which large amounts of intracellular GA were present in HK-2 cells. Although the amount of GA produced and accumulated in human kidney tissue in vivo may be lower, this has not yet been confirmed. Although we demonstrated that high concentrations of GA can induce cellular stress and cytotoxicity, the underlying mechanisms remain unclear. Although HMW immunoreactive species of HSP90β, Beclin-1, and ATG7 were detected, we were unable to distinguish cytotoxicity associated with these phenomena from cytotoxicity occurring independently of them.

## Data Availability

The data presented in this study are available from the corresponding author upon request.

## Supplementary Materials

The following supporting information can be downloaded at: https://www.mdpi.com/article/10.3390/biom16101488/s1, Figure S1: representative phase-contrast images of HK-2 cells treated with 0, 0.5, 1, 1.5, or 2 mM GA for 24 h. Figure S2: representative phase-contrast images of HK-2 cells treated with 0, 1.5, or 2 mM GA with or without AG pretreatment. Figure S3: full-length blots from three independent experiments examining HSP90 (HSP90α and HSP90β) with GA treatment. Figure S4: full-length blots from three independent experiments examining HSP90α with GA treatment. Figure S5: full-length blots from three independent experiments examining HSP90β with GA treatment. Figure S6: full-length blots from three independent experiments examining Beclin-1 with GA treatment. Figure S7: full-length blots from three independent experiments examining ATG7 with GA treatment. Figure S8: full-length blots from three independent experiments examining LC3 with GA treatment. Figure S9: full-length blots from three independent experiments examining p62 with GA treatment. Figure S10: full-length blots from three independent experiments examining HSP90β with GA treatment and AG pretreatment. Figure S11: full-length blots from three independent experiments examining Beclin-1 with GA treatment and AG pretreatment. Figure S12: full-length blots from three independent experiments examining ATG7 with GA treatment and AG pretreatment. Figure S13: full-length blots from three independent experiments examining LC3 with GA treatment and AG pretreatment. Figure S14: full-length blots from three independent experiments examining p62 with GA treatment and AG pretreatment.

## Abbreviations

AG	Aminoguanidine
AGEs	Advanced glycation end-products
ATP	Adenosine triphosphate
AU	Arbitrary units
BCA	Bicinchoninic acid
BSA	Bovine serum albumin
CKD	Chronic kidney disease
CML	*N*^ε^-carboxymethyl-lysine
DAB	3,3′-diaminobenzidine tetrahydrochloride
DN	Diabetic nephropathy
EDTA	Ethylenediamine-N,N,N′,N′-tetraacetic acid
FBS	Fetal bovine serum
GA	Glyceraldehyde
GA-AGEs	GA-derived advanced glycation end-products
GLAP	Glyceraldehyde-related pyridinium
H&E	Hematoxylin and eosin
HFCS	High-fructose corn syrup
HMW	High molecular weight
HSP90	Heat shock protein 90
LC3	Microtubule-associated protein 1 light chain 3
MG-H1	Methylglyoxal-derived hydroimidazolone
NADPH	Nicotinamide adenine dinucleotide phosphate
PBS	Phosphate-buffered saline
PTE	Proximal tubular epithelial
PVDF	Polyvinylidene fluoride
RIPA	Radioimmunoprecipitation assay
RT	Room temperature
SD	Standard deviation
SDS	Sodium dodecyl sulfate
STZ	Streptozotocin
TAGE	Toxic advanced glycation end-products
WB	Western blot
