# Supplementary material for: Glyceraldehyde May Induce the Generation of High-Molecular-Weight Species of HSP90β and Autophagy-Related Proteins and Alter Protein Levels in Human Renal Epithelial Cells
Source: Biomolecules. 2026 Oct 13;16(10):1488. doi: 10.3390/biom16101488 (PMC13702707; doi:10.3390/biom16101488)
Supplement: Supplementary file 1 [file biomolecules-16-01488-s001.zip › biomolecules-4591532-Supplementary Materials.pdf]

Supplementary Materials

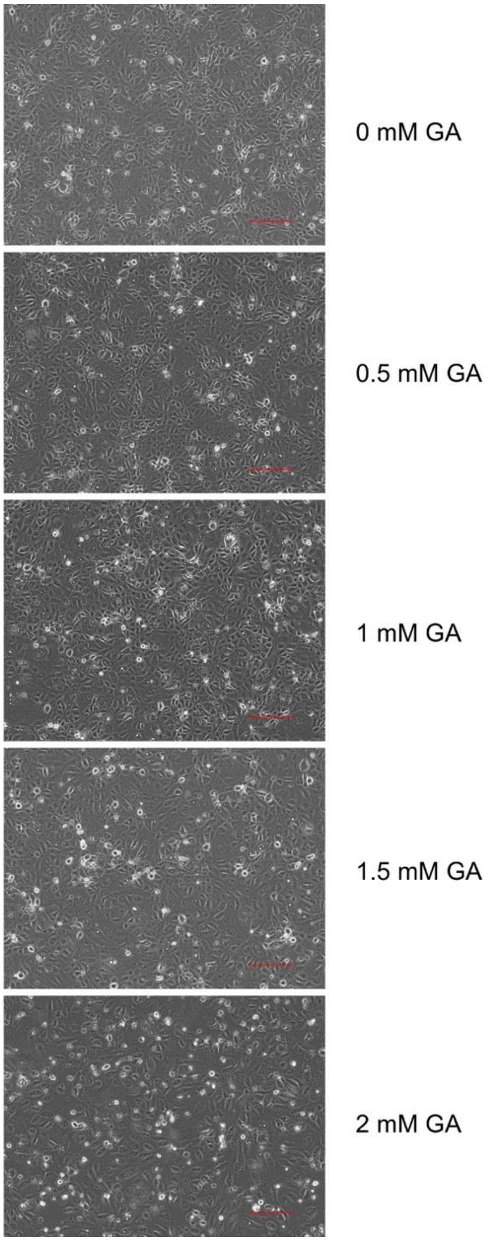

Figure S1. Representative phase-contrast images of HK-2 cells treated with 0, 0.5, 1, 1.5, and 2 mM glyceraldehyde (GA) for 24 h. Scale bars = 200 μm.

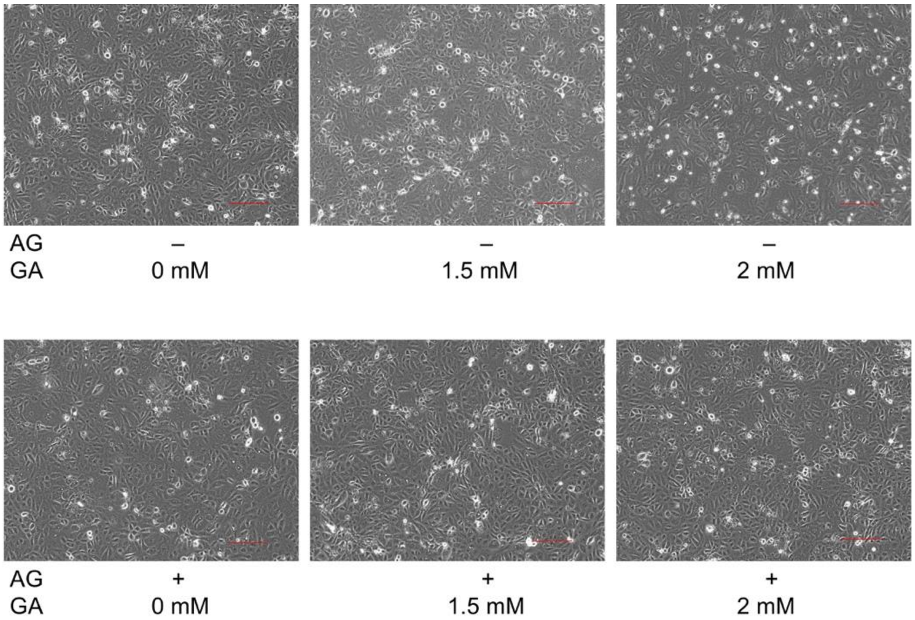

Figure S2. Representative phase-contrast images of HK-2 cells treated with 0, 1.5, and 2 mM GA with or without aminoguanidine (AG) pretreatment. Upper panels: without AG pretreatment. Lower panels: cells were pretreated with 8 mM AG for 2 h before treatment with the indicated concentrations of GA for 24 h. Scale bars = 200 μm.

(A)

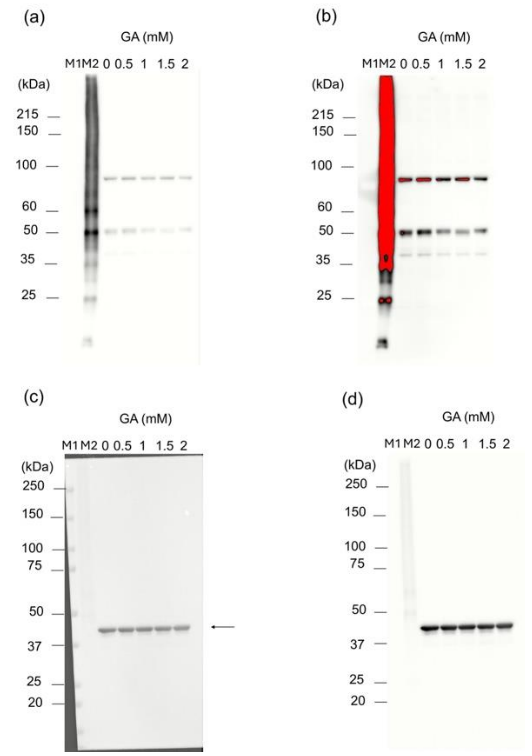
(a)
GA (mM)
M1M2 0 0.5 1 1.5 2
(kDa)
215
150
100
60
50
35
25
(b)
GA (mM)
M1M2 0 0.5 1 1.5 2
(kDa)
215
150
100
60
50
35
25
(c)
GA (mM)
M1M2 0 0.5 1 1.5 2
(kDa)
250
150
100
75
50
37
25
20
(d)
GA (mM)
M1 M2 0 0.5 1 1.5 2
(kDa)
250
150
100
75
50
37
25
20

(B)

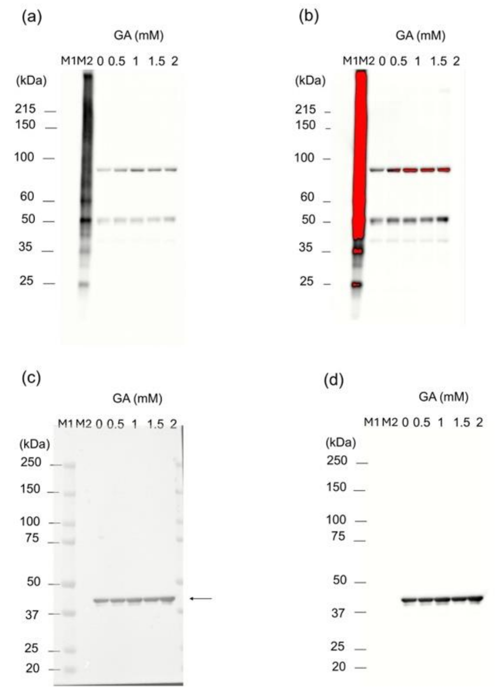
(a)
GA (mM)
M1M2 0 0.5 1 1.5 2
(kDa)
215
150
100
60
50
35
25
(b)
GA (mM)
M1M2 0 0.5 1 1.5 2
(kDa)
215
150
100
60
50
35
25
(c)
GA (mM)
M1 M2 0 0.5 1 1.5 2
(kDa)
250
150
100
75
50
37
25
20
(d)
GA (mM)
M1 M2 0 0.5 1 1.5 2
(kDa)
250
150
100
75
50
37
25
20

(C)

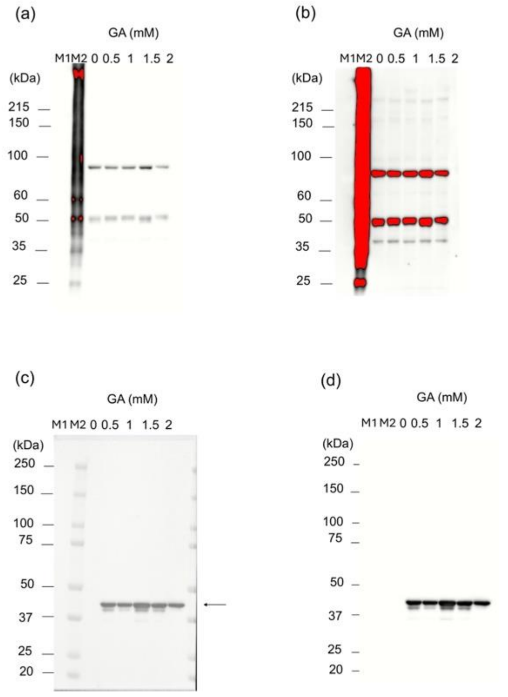

Figure S3. Full-length blots from three independent experiments examining heat shock protein 90 (HSP90α and HSP90β) with GA treatment.

(A) First independent experiment. **(a)** HSP90 band detected on polyvinylidene fluoride (PVDF) membrane with full sensitivity and exposure time of 40 s. **(b)** HSP90 band detected on PVDF membrane with full sensitivity and exposure time of 300 s. **(c)** PVDF membrane exposed for 360 ms with white Epi mode. The arrow indicates the position of compounds generated from anti-β-actin antibody, secondary antibody, and reagents of ImmunoStar LD kit. **(d)** β-actin band detected on PVDF membrane with full sensitivity and exposure time of 2 s. **(a–d)** M1: molecular weight size marker observable under visible light in this lane. M2: HRP-linked molecular weight size marker loaded in this lane. **(a, b)** Molecular weights determined based on the marker size on M2. **(c, d)** Molecular weights determined based on the marker size on M1.

(B) Second independent experiment

(C) Third independent experiment

(A)

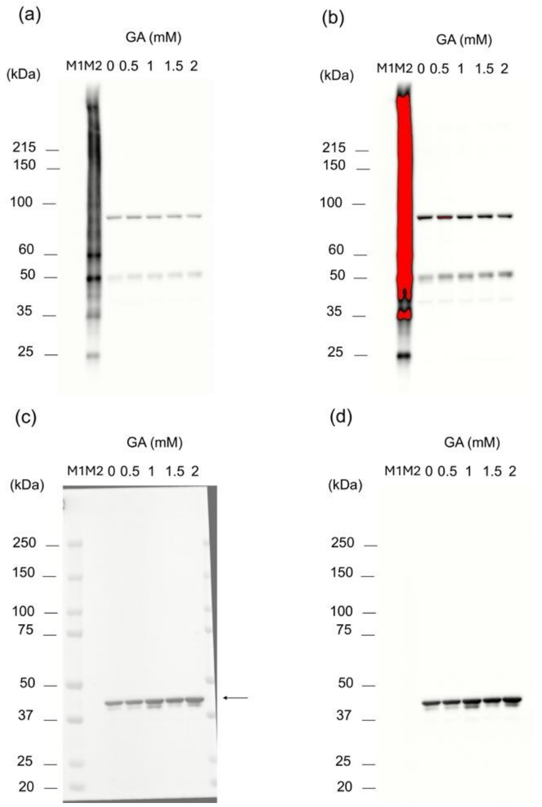
(a)
GA (mM)
M1M2 0 0.5 1 1.5 2
(kDa)
215
150
100
60
50
35
25
(b)
GA (mM)
M1M2 0 0.5 1 1.5 2
(kDa)
215
150
100
60
50
35
25
(c)
GA (mM)
M1M2 0 0.5 1 1.5 2
(kDa)
250
150
100
75
50
37
25
20
(d)
GA (mM)
M1M2 0 0.5 1 1.5 2
(kDa)
250
150
100
75
50
37
25
20

(B)

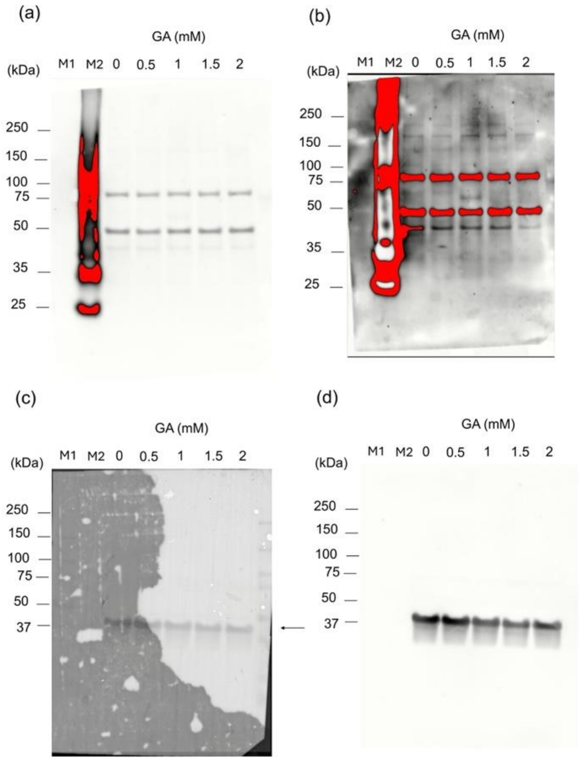
(a)
GA (mM)
M1 M2 0 0.5 1 1.5 2
(kDa)
250
150
100
75
50
35
25
(b)
GA (mM)
M1 M2 0 0.5 1 1.5 2
(kDa)
250
150
100
75
50
35
25
(c)
GA (mM)
M1 M2 0 0.5 1 1.5 2
(kDa)
250
150
100
75
50
37
(d)
GA (mM)
M1 M2 0 0.5 1 1.5 2
(kDa)
250
150
100
75
50
37

(C)

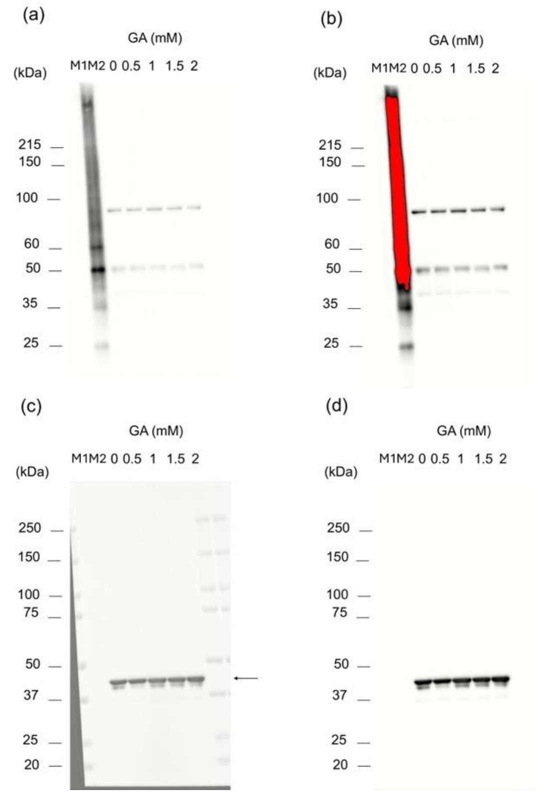

Figure S4. Full-length blots from three independent experiments examining HSP90α with GA treatment.

(A) First independent experiment, corresponding to Figure 3a, b. **(a)** HSP90α band detected on PVDF membrane with full sensitivity and exposure time of 15 s. **(b)** HSP90α band detected on PVDF membrane with full sensitivity and exposure time of 300 s. **(c)** PVDF membrane exposed for 360 ms with white Epi mode. The arrow indicates the position of compounds generated from anti-β-actin antibody, secondary antibody, and reagents of ImmunoStar LD kit. **(d)** β-actin band detected on PVDF membrane with full sensitivity and exposure time of 3 s. **(a–d)** M1: molecular weight size marker observable under visible light in this lane. M2: HRP-linked molecular weight size marker loaded in this lane. **(a, b)** Molecular weights determined based on the marker size on M2. **(c, d)** Molecular weights determined based on marker size on M1.

(B) Second independent experiment

(C) Third independent experiment

(A)

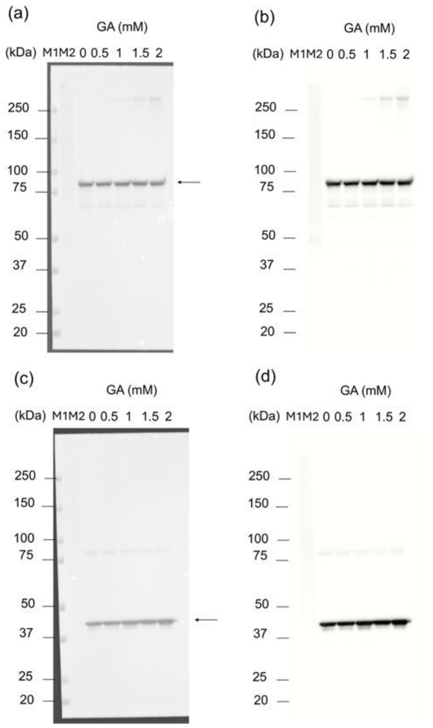
(a)
GA (mM)
(kDa) M1M2 0 0.5 1 1.5 2
250
150
100
75
50
37
25
20
(b)
GA (mM)
(kDa) M1M2 0 0.5 1 1.5 2
250
150
100
75
50
37
25
20
(c)
GA (mM)
(kDa) M1M2 0 0.5 1 1.5 2
250
150
100
75
50
37
25
20
(d)
GA (mM)
(kDa) M1M2 0 0.5 1 1.5 2
250
150
100
75
50
37
25
20

(B)

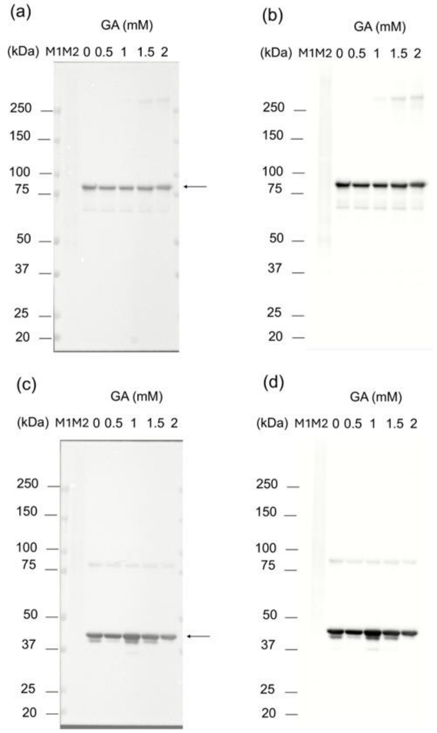
(a)
GA (mM)
(kDa) M1M2 0 0.5 1 1.5 2
250
150
100
75
50
37
25
20
(b)
GA (mM)
(kDa) M1M2 0 0.5 1 1.5 2
250
150
100
75
50
37
25
20
(c)
GA (mM)
(kDa) M1M2 0 0.5 1 1.5 2
250
150
100
75
50
37
25
20
(d)
GA (mM)
(kDa) M1M2 0 0.5 1 1.5 2
250
150
100
75
50
37
25
20

(C)

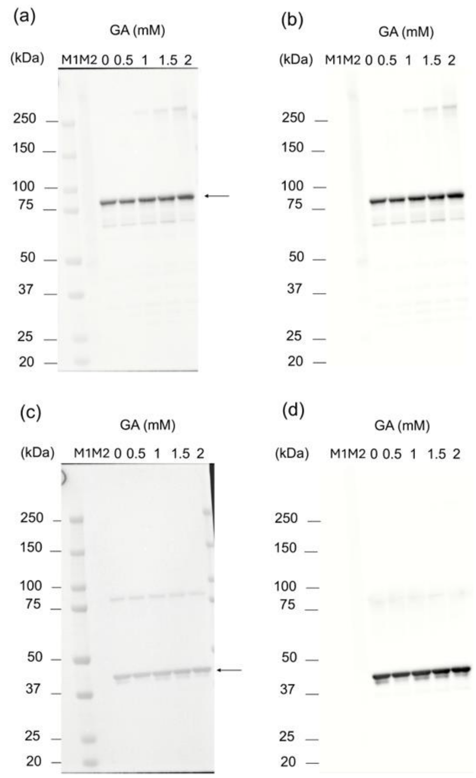

Figure S5. Full-length blots from three independent experiments examining HSP90β with GA treatment.

(A) First independent experiment, corresponding to Figure 4a. **(a)** PVDF membrane exposed for 360 ms with white Epi mode. The arrow indicates the position of HSP90β band. **(b)** HSP90β band detected on PVDF membrane with full sensitivity and exposure time of 10 s. **(c)** PVDF membrane exposed for 360 ms with white Epi mode. The arrow indicates the position of compounds generated from anti-β-actin antibody, secondary antibody, and reagents of ImmunoStar LD kit. **(d)** β-actin band detected on PVDF membrane with full sensitivity and exposure time of 7 s. **(a–d)** M1: molecular weight size marker observable under visible light in this lane. M2: HRP-linked molecular weight size marker loaded in this lane. The molecular weights are determined based on the marker size on M1.

(B) Second independent experiment

(C) Third independent experiment

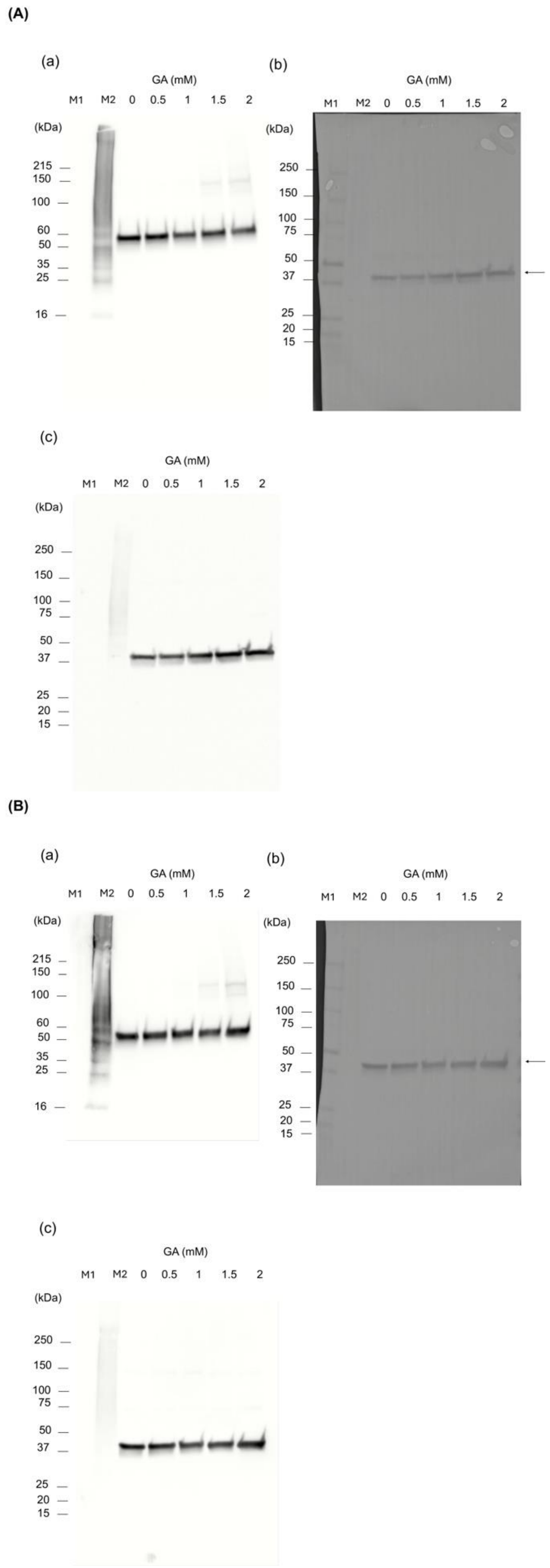
(A)
(a)
GA (mM)
M1 M2 0 0.5 1 1.5 2
(kDa)
215
150
100
60
50
35
25
16
(b)
GA (mM)
M1 M2 0 0.5 1 1.5 2
(kDa)
250
150
100
75
50
37
25
20
15
(c)
GA (mM)
M1 M2 0 0.5 1 1.5 2
(kDa)
250
150
100
75
50
37
25
20
15
(B)
(a)
GA (mM)
M1 M2 0 0.5 1 1.5 2
(kDa)
215
150
100
60
50
35
25
16
(b)
GA (mM)
M1 M2 0 0.5 1 1.5 2
(kDa)
250
150
100
75
50
37
25
20
15
(c)
GA (mM)
M1 M2 0 0.5 1 1.5 2
(kDa)
250
150
100
75
50
37
25
20
15

(C)

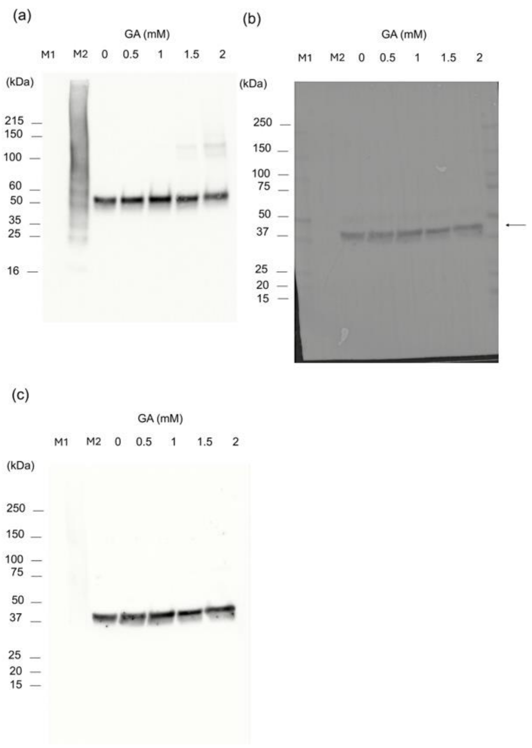

Figure S6. Full-length blots from three independent experiments examining Beclin-1 with GA treatment.

(A) First independent experiment, corresponding to Figure 5a. **(a)** Beclin-1 band detected on PVDF membrane with full sensitivity and exposure time of 12 s. **(b)** PVDF membrane exposed for 360 ms with white Epi mode. The arrow indicates the position of compounds generated from anti-β-actin antibody, secondary antibody, and reagents of ImmunoStar LD kit. **(c)** β-actin band detected on PVDF membrane with full sensitivity and exposure time of 10 s. **(a–c)** M1: molecular weight size marker observable under visible light in this lane. M2: HRP-linked molecular weight size marker loaded in this lane. **(a)** Molecular weights determined based on the marker size on M2. **(b, c)** Molecular weights determined based on the marker size on M1.

(B) Second independent experiment

(C) Third independent experiment

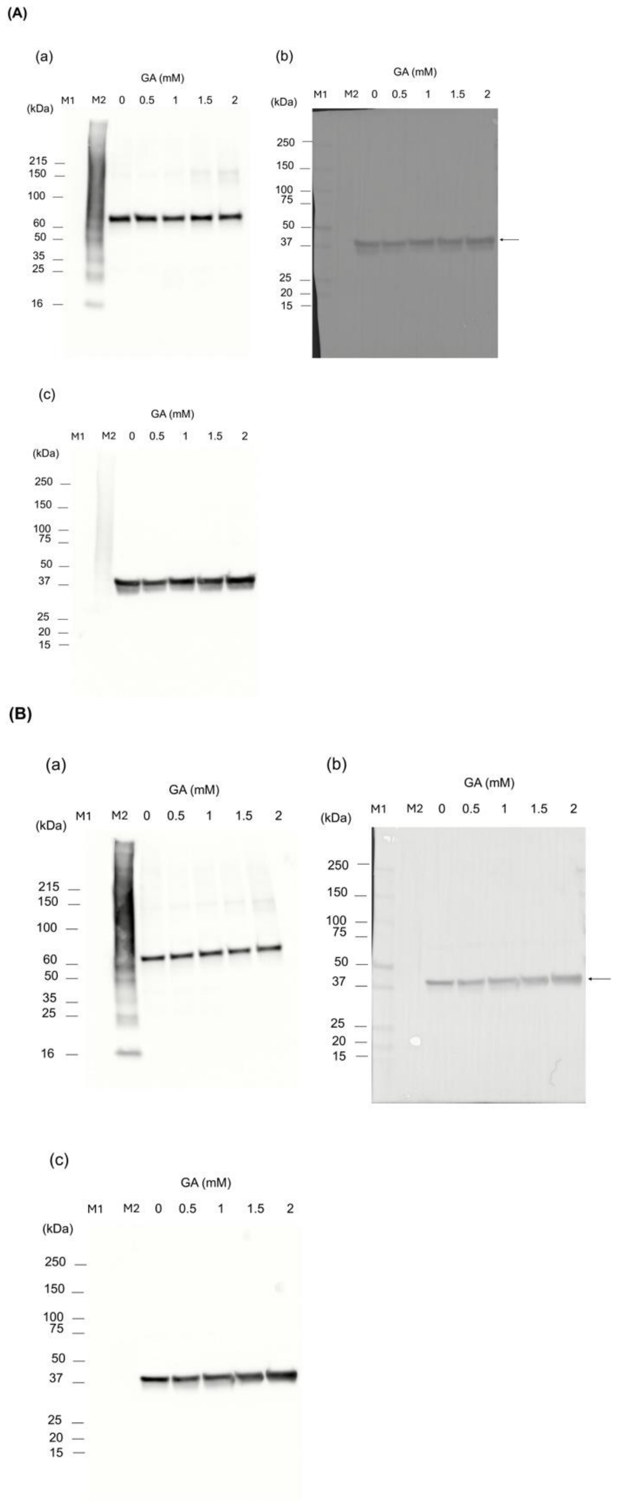
(A)
(a)
GA (mM)
M1 M2 0 0.5 1 1.5 2
(kDa)
215
150
100
60
50
35
25
16
(b)
GA (mM)
M1 M2 0 0.5 1 1.5 2
(kDa)
250
150
100
75
50
37
25
20
15
(c)
GA (mM)
M1 M2 0 0.5 1 1.5 2
(kDa)
250
150
100
75
50
37
25
20
15
(B)
(a)
GA (mM)
M1 M2 0 0.5 1 1.5 2
(kDa)
215
150
100
60
50
35
25
16
(b)
GA (mM)
M1 M2 0 0.5 1 1.5 2
(kDa)
250
150
100
75
50
37
25
20
15
(c)
GA (mM)
M1 M2 0 0.5 1 1.5 2
(kDa)
250
150
100
75
50
37
25
20
15

(C)

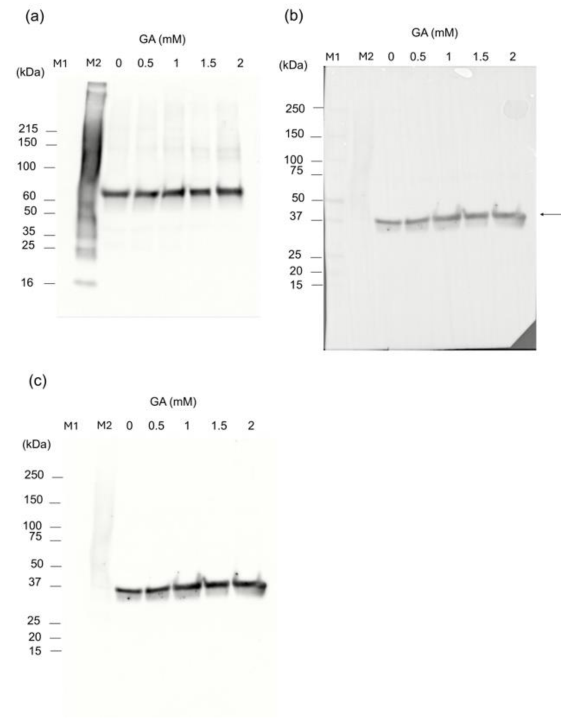

Figure S7. Full-length blots from three independent experiments examining ATG7 with GA treatment.

(A) First independent experiment, corresponding to Figure 6a. **(a)** ATG7 band detected on PVDF membrane with full sensitivity and exposure time of 10 s. **(b)** PVDF membrane exposed for 360 ms with white Epi mode. The arrow indicates the position of compounds generated from anti-β-actin antibody, secondary antibody, and reagents of ImmunoStar LD kit. **(c)** β-actin band detected on PVDF membrane with full sensitivity and exposure time of 5 s. **(a–c)** M1: molecular weight size marker observable under visible light in this lane. M2: HRP-linked molecular weight size marker loaded in this lane. **(a)** Molecular weights determined based on the marker size on M2. **(b, c)** Molecular weights determined based on the marker size on M1.

(B) Second independent experiment

(C) Third independent experiment

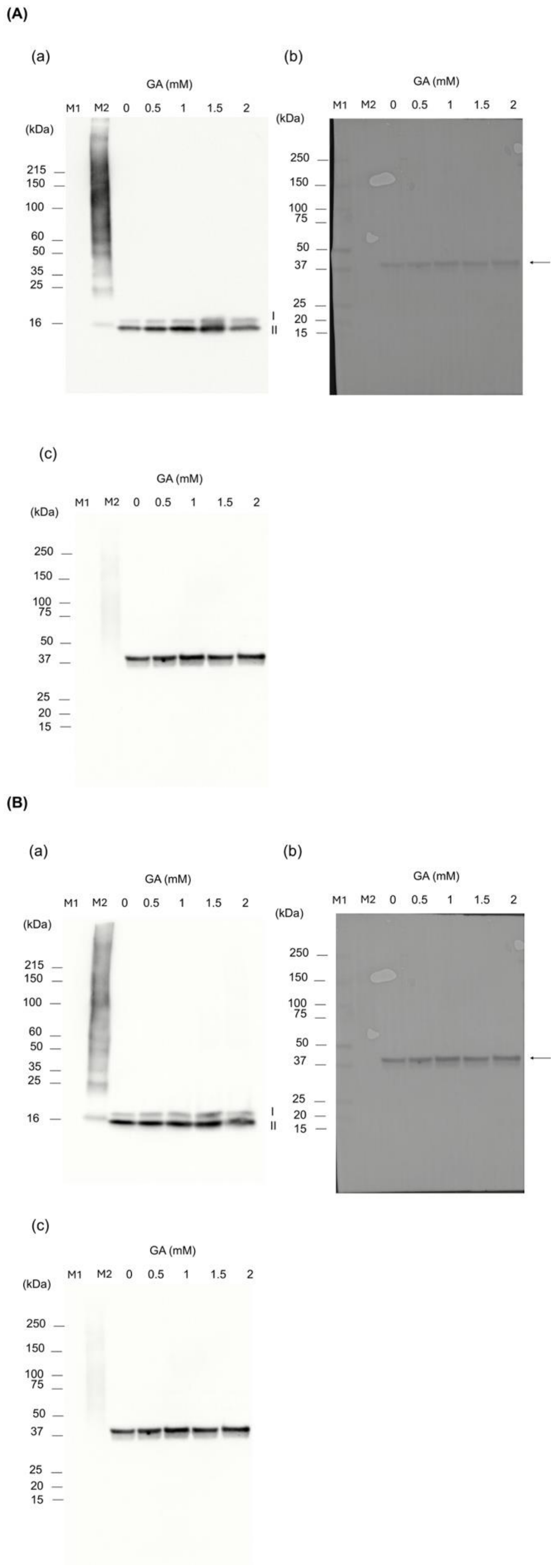
(A)
(a)
GA (mM)
M1 M2 0 0.5 1 1.5 2
(kDa)
215
150
100
60
50
35
25
16
I
II
(b)
GA (mM)
M1 M2 0 0.5 1 1.5 2
(kDa)
250
150
100
75
50
37
25
20
15
(c)
GA (mM)
M1 M2 0 0.5 1 1.5 2
(kDa)
250
150
100
75
50
37
25
20
15
(B)
(a)
GA (mM)
M1 M2 0 0.5 1 1.5 2
(kDa)
215
150
100
60
50
35
25
16
I
II
(b)
GA (mM)
M1 M2 0 0.5 1 1.5 2
(kDa)
250
150
100
75
50
37
25
20
15
(c)
GA (mM)
M1 M2 0 0.5 1 1.5 2
(kDa)
250
150
100
75
50
37
25
20
15

(C)

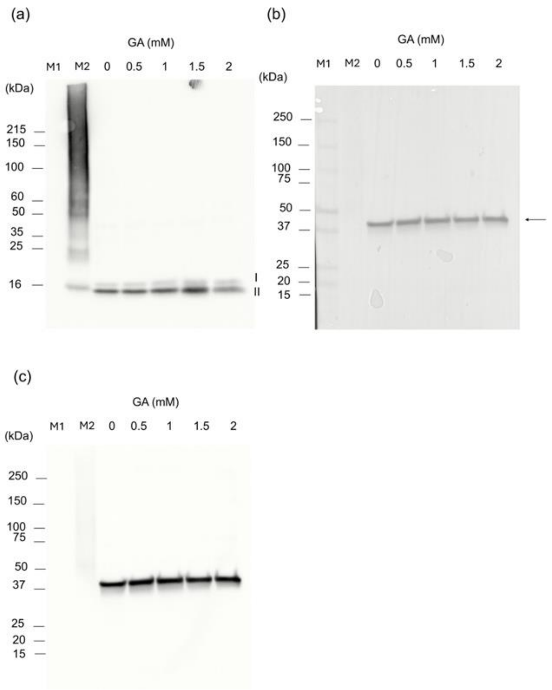

Figure S8. Full-length blots from three independent experiments examining microtubule-associated protein 1 light chain 3 (LC3) with GA treatment.

(A) First independent experiment, corresponding to Figure 7a. **(a)** LC3 band detected on PVDF membrane with full sensitivity and exposure time of 10 s. The positions of LC3-I and LC3-II are indicated by I and II. **(b)** PVDF membrane exposed for 360 ms with white Epi mode. The arrow indicates the position of compounds generated from anti-β-actin antibody, secondary antibody, and reagents of ImmunoStar LD kit. **(c)** β-actin band detected on PVDF membrane with full sensitivity and exposure time of 5 s. **(a–c)** M1: molecular weight size marker observable under visible light in this lane. M2: HRP-linked molecular weight size marker loaded in this lane. **(a)** Molecular weights determined based on the marker size on M2. **(b, c)** Molecular weights determined based on the marker size on M1.

(B) Second independent experiment

(C) Third independent experiment

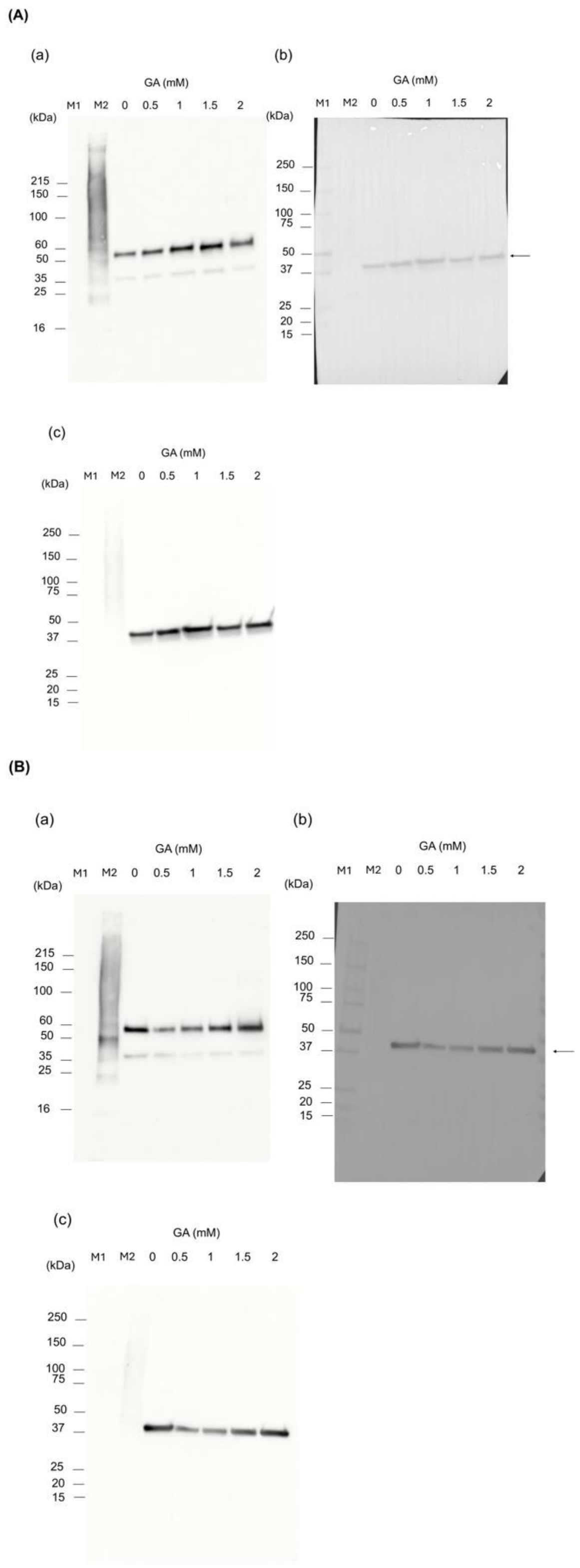

(A)
(a)
GA (mM)
M1 M2 0 0.5 1 1.5 2
(kDa)
215
150
100
60
50
35
25
16
(b)
GA (mM)
M1 M2 0 0.5 1 1.5 2
(kDa)
250
150
100
75
50
37
25
20
15
(c)
GA (mM)
M1 M2 0 0.5 1 1.5 2
(kDa)
250
150
100
75
50
37
25
20
15
(B)
(a)
GA (mM)
M1 M2 0 0.5 1 1.5 2
(kDa)
215
150
100
60
50
35
25
16
(b)
GA (mM)
M1 M2 0 0.5 1 1.5 2
(kDa)
250
150
100
75
50
37
25
20
15
(c)
GA (mM)
M1 M2 0 0.5 1 1.5 2
(kDa)
250
150
100
75
50
37
25
20
15

(C)

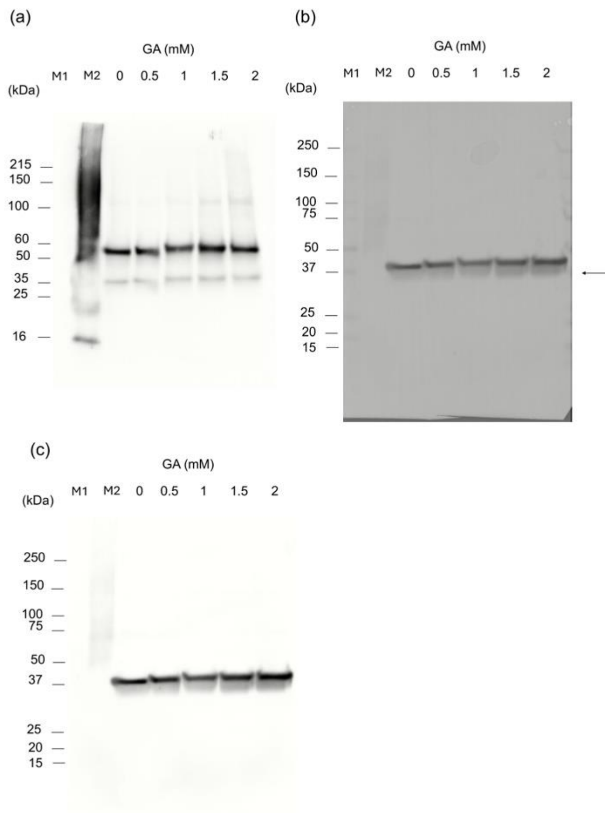

Figure S9. Full-length blots from three independent experiments examining p62 with GA treatment.

(A) First independent experiment, corresponding to Figure 8a. **(a)** p62 band detected on PVDF membrane with full sensitivity and exposure time of 5 s. **(b)** PVDF membrane exposed for 360 ms with white Epi mode. The arrow indicates the position of compounds generated from anti-β-actin antibody, secondary antibody, and reagents of ImmunoStar LD kit. **(c)** β-actin band detected on PVDF membrane with full sensitivity and exposure time of 5 s. **(a–c)** M1: molecular weight size marker observable under visible light in this lane. M2: HRP-linked molecular weight size marker loaded in this lane. **(a)** Molecular weights determined based on the marker size on M2. **(b, c)** Molecular weights determined based on the marker size on M1.

(B) Second independent experiment

(C) Third independent experiment

(A)

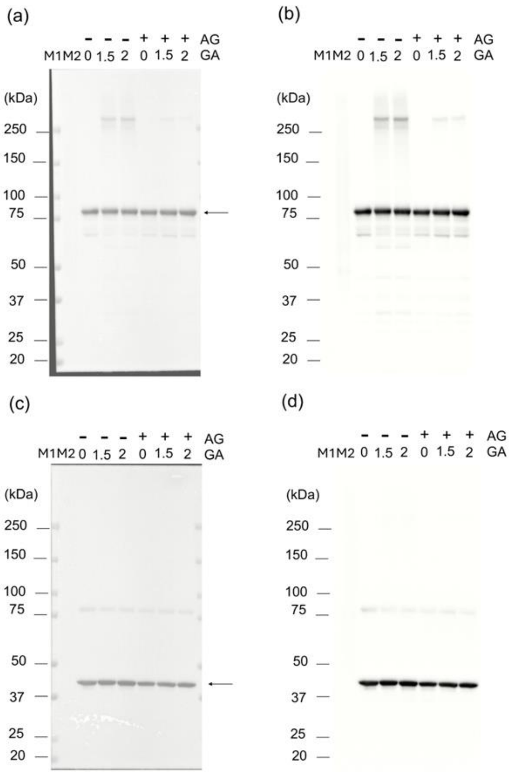

(a)
(b)
(c)
(d)
M1M2
0 1.5 2 0 1.5 2
AG
GA
(kDa)
250
150
100
75
50
37
25
20

(B)

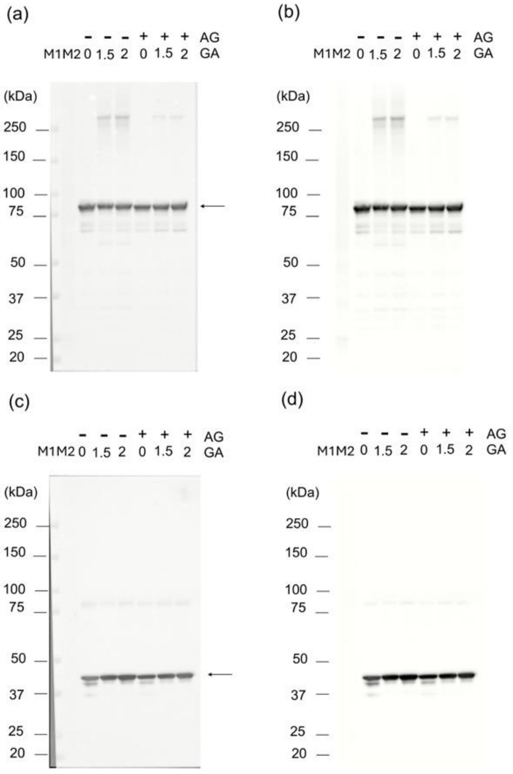

(a)
(b)
(c)
(d)
M1M2
0 1.5 2 0 1.5 2
AG
GA
(kDa)
250
150
100
75
50
37
25
20

(C)

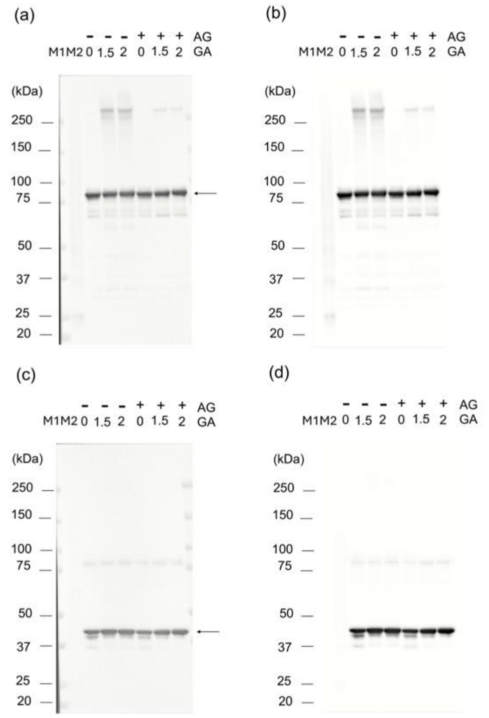

Figure S10. Full-length blots from three independent experiments examining HSP90β with GA treatment and AG pretreatment.

(A) First independent experiment, corresponding to Figure 10a. **(a)** PVDF membrane exposed for 360 ms with white Epi mode. The arrow indicates the position of HSP90β band. **(b)** HSP90β band detected on PVDF membrane with full sensitivity and exposure time of 12 s. **(c)** PVDF membrane exposed for 360 ms with white Epi mode. The arrow indicates the position of compounds generated from anti-β-actin antibody, secondary antibody, and reagents of ImmunoStar LD kit. **(d)** β-actin band detected on PVDF membrane with full sensitivity and exposure time of 5 s. **(a–d)** M1: molecular weight size marker observable under visible light in this lane. M2: HRP-linked molecular weight size marker loaded in this lane. The molecular weights are determined based on the marker size on M1.

(B) Second independent experiment

(C) Third independent experiment

(A)

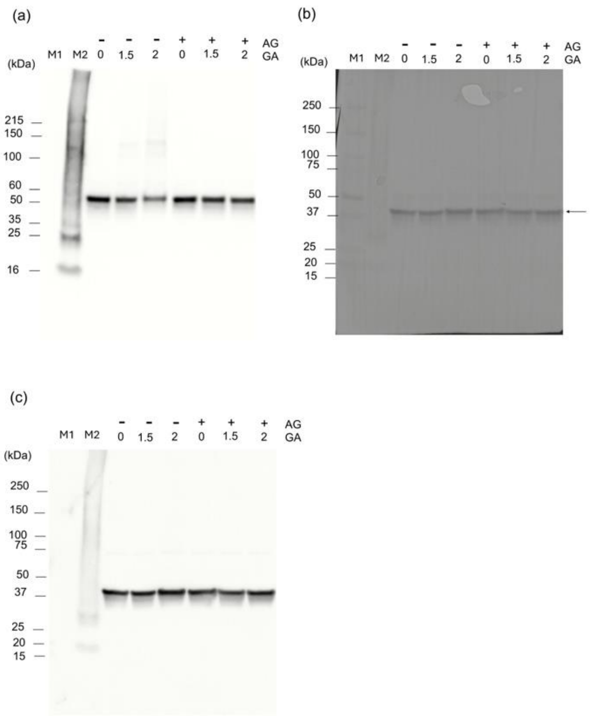
(a)
M1 M2 0 1.5 2 0 1.5 2 AG GA
(kDa)
215
150
100
60
50
35
25
16
(b)
M1 M2 0 1.5 2 0 1.5 2 AG GA
(kDa)
250
150
100
75
50
37
25
20
15
(c)
M1 M2 0 1.5 2 0 1.5 2 AG GA
(kDa)
250
150
100
75
50
37
25
20
15

(B)

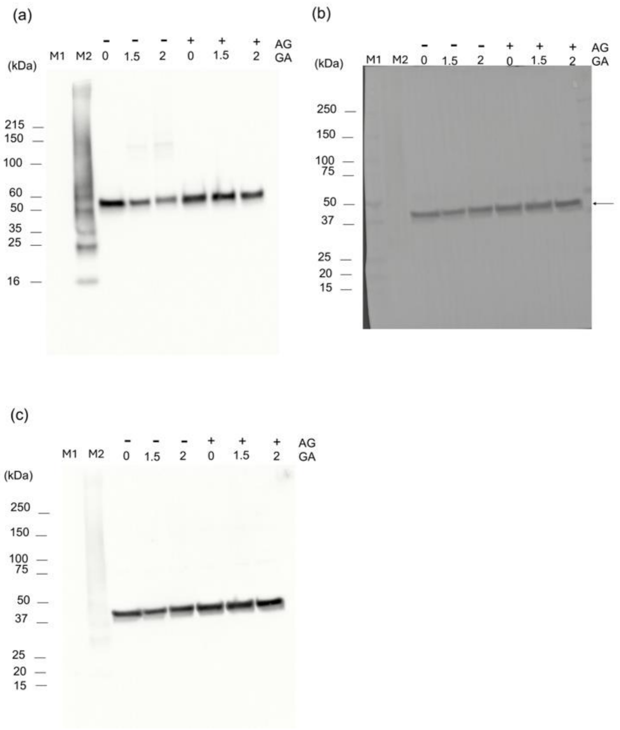
(a)
M1 M2 0 1.5 2 0 1.5 2 AG GA
(kDa)
215
150
100
60
50
35
25
16
(b)
M1 M2 0 1.5 2 0 1.5 2 AG GA
(kDa)
250
150
100
75
50
37
25
20
15
(c)
M1 M2 0 1.5 2 0 1.5 2 AG GA
(kDa)
250
150
100
75
50
37
25
20
15

(C)

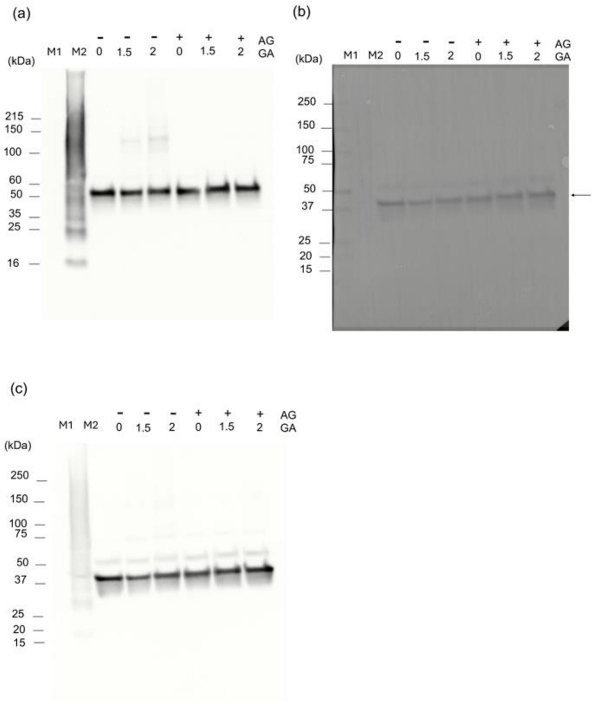

Figure S11. Full-length blots from three independent experiments examining Beclin-1 with GA treatment and AG pretreatment.

(A) First independent experiment, corresponding to Figure 11a. **(a)** Beclin-1 band detected on PVDF membrane with full sensitivity and exposure time of 8 s. **(b)** PVDF membrane exposed for 360 ms with white Epi mode. The arrow indicates the position of compounds generated from anti-β-actin antibody, secondary antibody, and reagents of ImmunoStar LD kit. **(c)** β-actin band detected on PVDF membrane with full sensitivity and exposure time of 5 s. **(a–c)** M1: molecular weight size marker observable under visible light in this lane. M2: HRP-linked molecular weight size marker loaded in this lane. **(a)** Molecular weights determined based on the marker size on M2. **(b, c)** Molecular weights determined based on the marker size on M1.

(B) Second independent experiment

(C) Third independent experiment

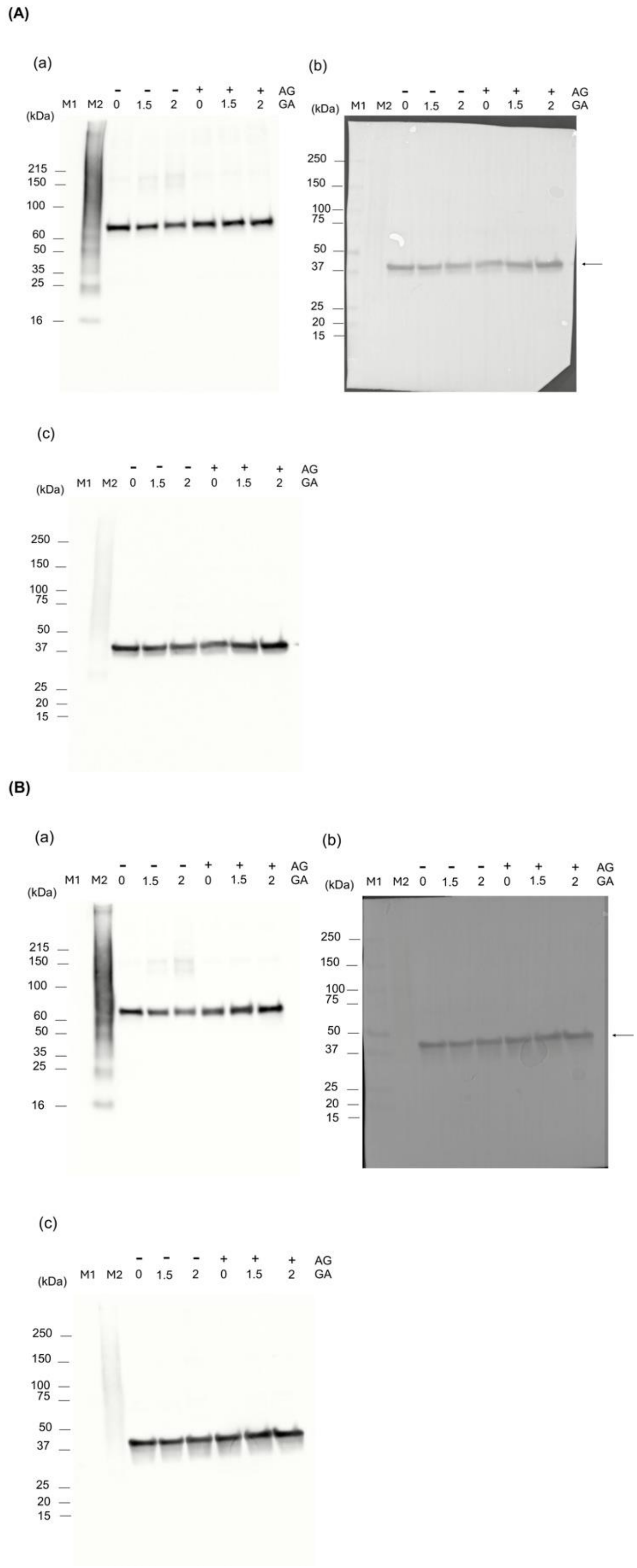
(A)
(a)
(b)
(c)
(B)
M1 M2
AG
GA
(kDa)
0 1.5 2
215
150
100
60
50
35
25
16
250
75
37
20
15

(C)

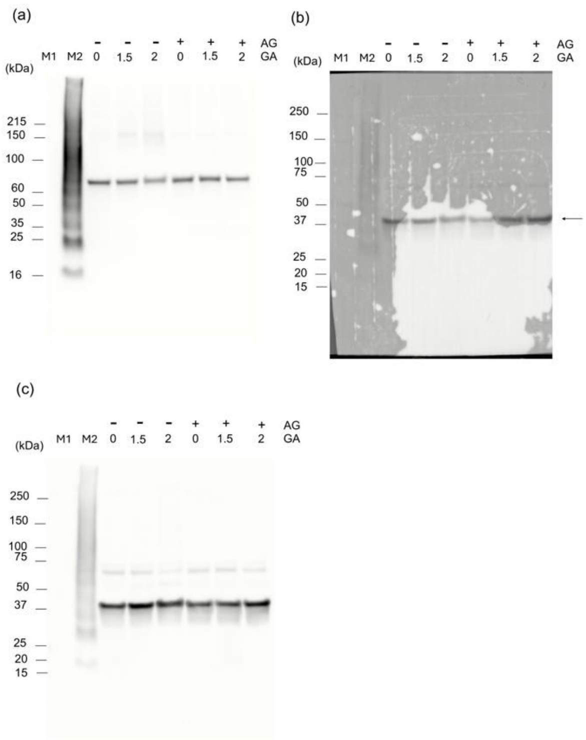

Figure S12. Full-length blots from three independent experiments examining ATG7 with GA treatment and AG pretreatment.

(A) First independent experiment, corresponding to Figure 12a. **(a)** ATG7 band detected on PVDF membrane with full sensitivity and exposure time of 5 s. **(b)** PVDF membrane exposed for 360 ms with white Epi mode. The arrow indicates the position of compounds generated from anti-β-actin antibody, secondary antibody, and reagents of ImmunoStar LD kit. **(c)** β-actin band detected on PVDF membrane with full sensitivity and exposure time of 10 s. **(a–c)** M1: molecular weight size marker observable under visible light in this lane. M2: HRP-linked molecular weight size marker loaded in this lane. **(a)** Molecular weights determined based on the marker size on M2. **(b, c)** Molecular weights determined based on the marker size on M1.

(B) Second independent experiment

(C) Third independent experiment

(A)

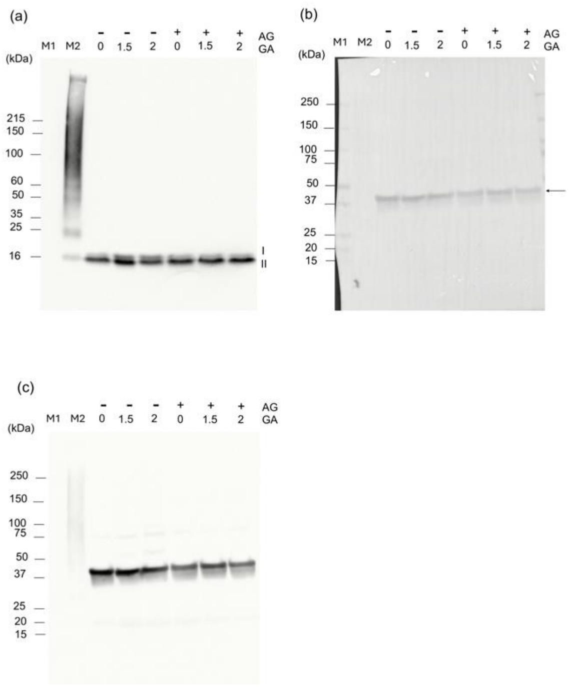
(a)
M1 M2 − 0 − 1.5 − 2 + 0 + 1.5 + 2 AG GA
(kDa)
215
150
100
60
50
35
25
16
I
II
(b)
M1 M2 − 0 − 1.5 − 2 + 0 + 1.5 + 2 AG GA
(kDa)
250
150
100
75
50
37
25
20
15
(c)
M1 M2 − 0 − 1.5 − 2 + 0 + 1.5 + 2 AG GA
(kDa)
250
150
100
75
50
37
25
20
15

(B)

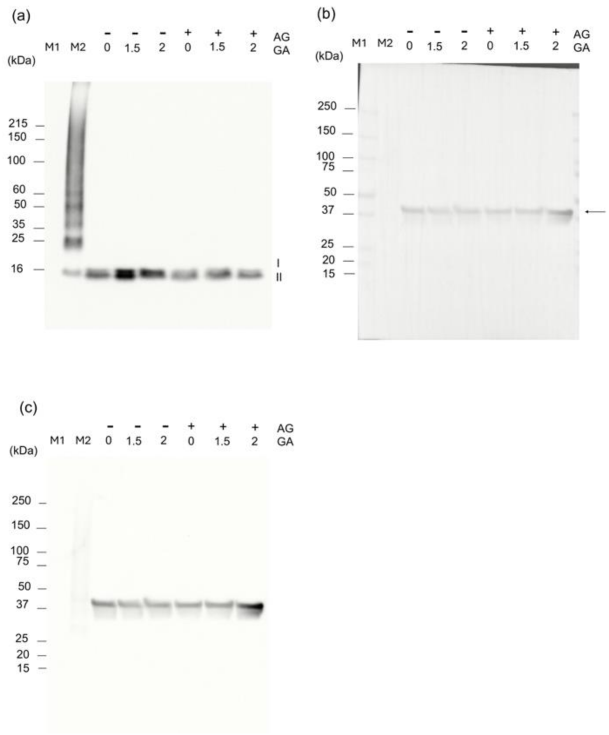
(a)
M1 M2 − 0 − 1.5 − 2 + 0 + 1.5 + 2 AG GA
(kDa)
215
150
100
60
50
35
25
16
I
II
(b)
M1 M2 − 0 − 1.5 − 2 + 0 + 1.5 + 2 AG GA
(kDa)
250
150
100
75
50
37
25
20
15
(c)
M1 M2 − 0 − 1.5 − 2 + 0 + 1.5 + 2 AG GA
(kDa)
250
150
100
75
50
37
25
20
15

(C)

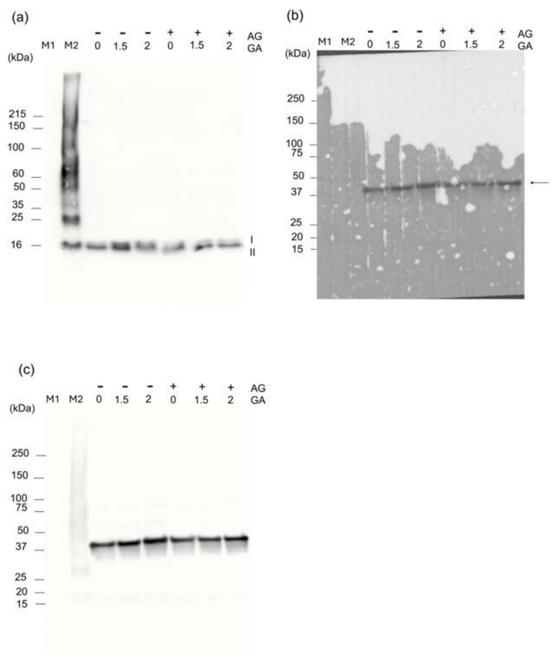

Figure S13. Full-length blots from three independent experiments examining LC3 with GA treatment and AG pretreatment.

(A) First independent experiment, corresponding to Figure 13a. **(a)** LC3 band detected on PVDF membrane with full sensitivity and exposure time of 8 s. The positions of LC3-I and LC3-II are indicated by I and II. **(b)** PVDF membrane exposed for 360 ms with white Epi mode. The arrow indicates the position of compounds generated from anti-β-actin antibody, secondary antibody, and reagents of ImmunoStar LD kit. **(c)** β-actin band detected on PVDF membrane with full sensitivity and exposure time of 6 s. **(a–c)** M1: molecular weight size marker observable under visible light in this lane. M2: HRP-linked molecular weight size marker loaded in this lane. **(a)** Molecular weights determined based on the marker size on M2. **(b, c)** Molecular weights determined based on the marker size on M1.

(B) Second independent experiment

(C) Third independent experiment

(A)

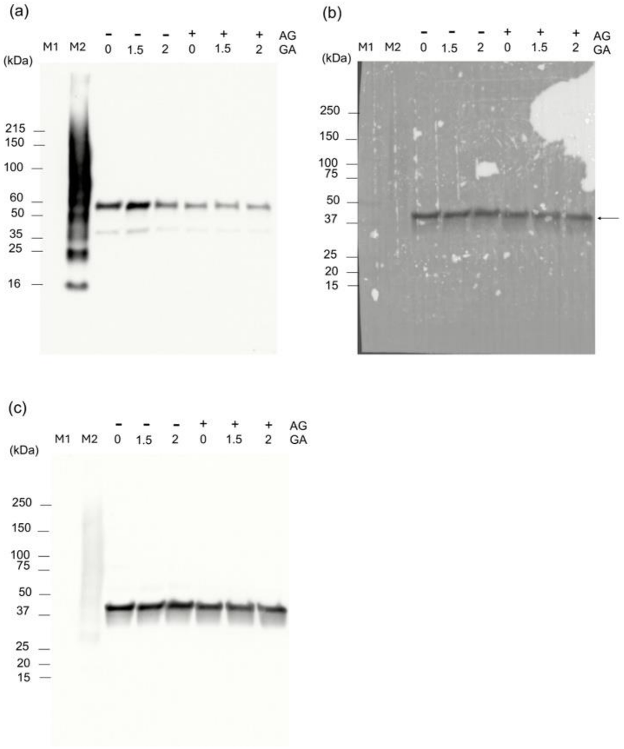
(a)
M1 M2
− − − + + + AG
0 1.5 2 0 1.5 2 GA
(kDa)
215
150
100
60
50
35
25
16
(b)
M1 M2
− − − + + + AG
0 1.5 2 0 1.5 2 GA
(kDa)
250
150
100
75
50
37
25
20
15
(c)
M1 M2
− − − + + + AG
0 1.5 2 0 1.5 2 GA
(kDa)
250
150
100
75
50
37
25
20
15

(B)

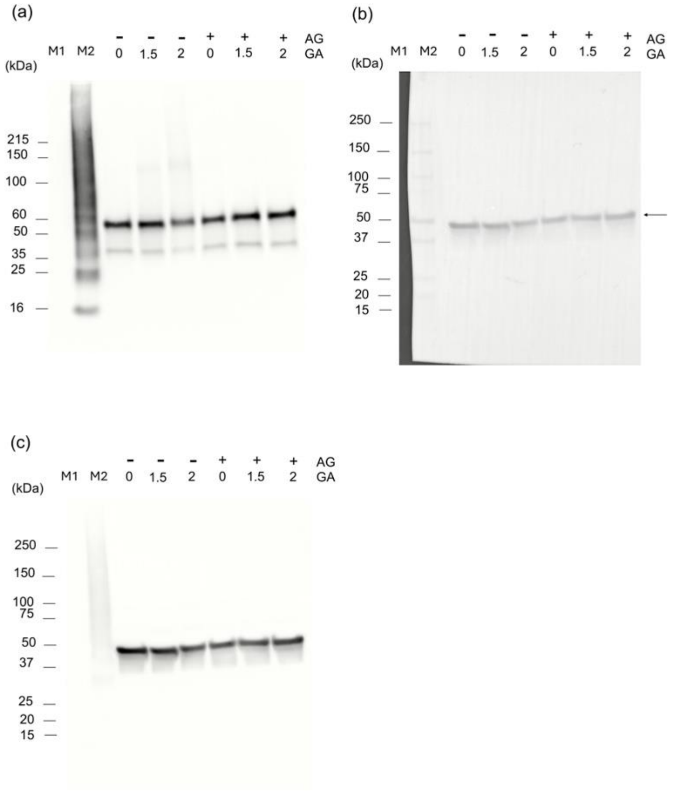
(a)
M1 M2
− − − + + + AG
0 1.5 2 0 1.5 2 GA
(kDa)
215
150
100
60
50
35
25
16
(b)
M1 M2
− − − + + + AG
0 1.5 2 0 1.5 2 GA
(kDa)
250
150
100
75
50
37
25
20
15
(c)
M1 M2
− − − + + + AG
0 1.5 2 0 1.5 2 GA
(kDa)
250
150
100
75
50
37
25
20
15

(C)

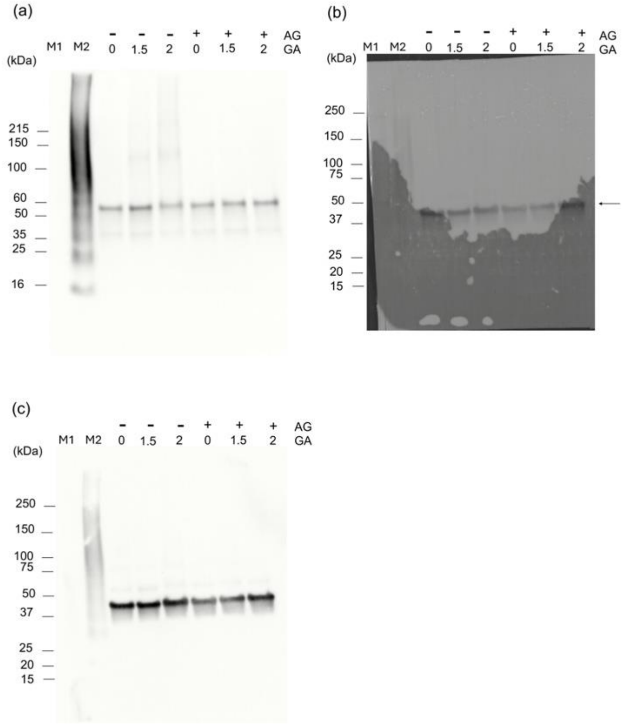

Figure S14. Full-length blots from three independent experiments examining p62 with GA treatment and AG pretreatment.

(A) First independent experiment, corresponding to Figure 14a. **(a)** p62 band detected on PVDF membrane with full sensitivity and exposure time of 20 s. **(b)** PVDF membrane exposed for 360 ms with white Epi mode. The arrow indicates the position of compounds generated from anti-β-actin antibody, secondary antibody, and reagents of ImmunoStar LD kit. **(c)** β-actin band detected on PVDF membrane with full sensitivity and exposure time of 5.5 s. **(a–c)** M1: molecular weight size marker observable under visible light in this lane. M2: HRP-linked molecular weight size marker loaded in this lane. **(a)** Molecular weights determined based on the marker size on M2. **(b, c)** Molecular weights determined based on the marker size on M1.

(B) Second independent experiment

(C) Third independent experiment
